# Keratinocytes biocompatibility, antibacterial and antioxidant properties of porous coacervate phosphate glass fibres and powders loaded with cerium and clove oil: a comparative study

**DOI:** 10.1039/d5tb02254a

**Published:** 2026-03-30

**Authors:** Zarrin Moghaddam, Rahul Sanwlani, Eveliny Tomás Nery, Irem Unalan, Oluwadunmininu Okude, Agron Hoxha, Charlotte A. Berry, Kavin Hettiarachchilage, Steven J. Hinder, Mark A. Baker, Monica Felipe-Sotelo, Alessandra Pinna, Jorge Merino-Gutierrez, Aldo R. Boccaccini, Patrizia Camelliti, Daniela Carta

**Affiliations:** a School of Chemistry and Chemical Engineering, University of Surrey Guildford GU2 7XH UK d.carta@surrey.ac.uk; b School of Biosciences, University of Surrey Guildford GU2 7XH UK; c Institute of Biomaterials, Department of Materials Science and Engineering, Friedrich-Alexander-University Erlangen-Nuremberg Caustraße 6 91058 Erlangen Germany; d School of Veterinary Medicine, Faculty of Health and Medical Sciences, University of Surrey Guildford GU2 7XH UK; e The Surface Analysis Laboratory, Engineering and Physical Sciences, University of Surrey GU2 7XH Guildford UK; f Department of Materials, Imperial College London SW7 2AZ London UK

## Abstract

Porous biomaterials offer significant advantages in tissue engineering by promoting cellular attachment and enhancing flow of fluids. Here we present a versatile, sustainable and low-cost manufacturing technology to produce porous phosphate-based glass fibres (PGFs) and powders (PGPs) in the system P_2_O_5_–CaO–Na_2_O unloaded and loaded with cerium ions (Ce = 0, 0.1, 0.2 and 0.4 atomic%). A polyphosphate coacervate gel combined with the porogen Pluronic 123 (P123) was used as precursor material for manufacturing PGFs (*via* electrospinning) and PGPs (*via* drying). Porosity was achieved by removing P123 *via* calcination at 300 °C. Cerium loaded PGPs and PGFs showed good antibacterial activity against the bacterium *E. coli*. The oxidation state of cerium ions was identified *via* X-ray Photoelectron Spectroscopy (XPS). Successful direct seeding of keratinocyte cells (HaCaTs) on PGFs was demonstrated for the first time *via* live cell staining. PGFs’ dissolution products also exhibited excellent cytocompatibility, demonstrating the great potential of PGFs for wound healing applications. To enhance the antibacterial and antioxidant properties, PGFs and PGPs were embedded with the natural antioxidant clove oil (clv). The antioxidant capacity was evaluated using DPPH (2,2-diphenyl-1-picrylhydrazyl) radical scavenging and TPC (Total Phenolic Content) assays. PGFs containing 0.4 atomic% of cerium loaded with clv demonstrated strong antioxidant activity, with DPPH scavenging reaching approximately 87% and phenolic content of around 25 mg gallic acid (GAE) g^−1^. Finally, the combined effects of cerium ions and clv were further assessed against *E. coli* and in cell-based assays, including intracellular ROS and scratch wound healing tests. PG-unl-clv and PG-Ce0.2-clv scavenged intracellular ROS and achieved significant wound closure after 48 h.

## Abstract 2

Porous coacervate phosphate glasses fibres and powders loaded with gallium and clove oil for wound healing applications.

## Graphical abstract

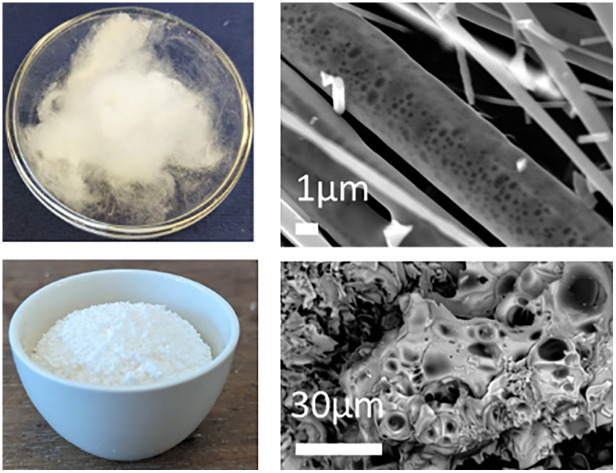

## 1. Introduction

The field of porous biomaterials for tissue engineering is rapidly expanding.^1^ Porosity enhances biomaterial's performance by facilitating transport of fluids and nutrients, promoting interactions with cells, and enhancing tissue ingrowth and vascularization.^2^ Porosity also allows higher loading capacity and absorption of therapeutic molecules such as drugs, antibacterial ions and antioxidant species (*e.g.* essential oils). Porous materials can find applications in both hard and soft tissue regeneration as they can be tailored to mimic the structure of the host tissue.

In hard tissue regeneration, interconnected porosity within scaffolds creates space for essential cellular activities, including adhesion, migration, proliferation, and differentiation.^3^ Large pores, known as macropores (>50 nm), are particularly effective in supporting the migration and infiltration of key cells like osteoblasts and mesenchymal stem cells, which are essential for bone formation.^1^ In the context of soft tissue regeneration, porous constructs improve nutrient and oxygen delivery, thereby supporting vascular integration, one of the biggest challenges in the formulation of matrices for skin regeneration.^1^

Among porous biomaterials, porous silicate-based glasses have been extensively studied as materials for tissue regeneration. Thanks to their peculiar textural properties and bioactivity, they show enhanced bone bonding ability, excellent cell ingrowth and integration with surrounding tissues.^5,6^ However, their relatively slow degradation and limited bioresorbability can restrict full replacement by newly formed tissue. Moreover, their long-term reaction in the body is still unknown.^7^ Phosphate-based glasses (PGs) have been presented as a promising class of bioactive materials alternative to silicate-based systems.^8^ Being bioresorbable, PGs gradually degrade while releasing therapeutic agents in a controlled manner, ultimately being replaced by newly formed tissue.

PGs present advantages over borate-based glasses too whose potential toxicity due to the release of borate ions is an issue. Ions released from PGs already exist in the body, causing no toxicity or biocompatibility issues. Moreover, most of borate glasses used in biomedicine are produced by the melt-quenching (MQ) technique which requires high temperatures (>1000 °C). This does not allow the incorporation of temperature sensitive and can cause reduction of antimicrobial ions and compositional changes due to loss of volatiles.

The most common PGs used as biomaterials are derived from the ternary system P_2_O_5_–CaO–Na_2_O, often incorporating therapeutic species such as Cu, Ag, Ga, and Ce ions, which imbue PGs with antibacterial and/or antioxidant properties. PGs are commonly produced *via* MQ which makes the manufacturing of porous PGs very challenging.^9^ The sol–gel (SG) method, considered as an in-solution alternative to the MQ process, has been used for the synthesis of porous silicate-based glasses. However, unlike the silicate systems, porous PGs are more challenging to be produced *via* the SG method. This is because the phosphate network is far more susceptible to structural collapse and crystallization than the silicate one.^10,11^ Only very recently, Foroutan *et al.* have presented mesoporous PGs (MPGs) prepared *via* the SG technique with enhanced drug loading efficiency and release compared to the corresponding non-porous PGs systems.^12–14^ However, the SG method requires the use of organic solvents and can be time consuming.^9^

The coacervation method, an in-solution route alternative to the SG method, has been recently used for the synthesis of non-porous PGs.^15^ Coacervation allows fabrication of PGs in both powder and fibre form using the same gel precursor, at room temperature and in aqueous solution. The simple, versatile and sustainable manufacturing process of coacervation avoids the use of organic solvents and reduces the manufacturing temperature with a positive impact on biocompatibility and on the environment.^16^ This technique consists of gradually adding a M^2+^ ion (M = Ca, Mg, Sr) to an aqueous solution of sodium polyphosphate under continuous stirring; this causes a phase separation between an aqueous supernatant and a bottom gel-like phase (coacervate). The coacervate gel, isolated from the supernatant is then vacuum dried to obtain PGPs or drawn into fibres *via* electrospinning (ES) to obtain PGFs.

The manufacturing process of coacervation is particularly interesting for the synthesis of PGFs *via* ES as it allows introduction of porosity within the fibres (intra-porosity) in addition to the inter-porosity already present in between the fibres.

Porous fibres are particularly indicated for soft tissue regeneration, given that their morphology is similar to the extracellular matrix (ECM);^18^ however, they can also find application in hard tissue regeneration, being easily packed into complex bone defects.^17^

In this work, the coacervation technique combined with supramolecular templating was used for producing PGPs and PGFs in the system P_2_O_5_–NaO–CaO–Ce*x*O*y* (Ce = 0, 0.1, 0.2 and 0.4 atomic%).

Cerium ions were incorporated into the system due to their reported antibacterial, antioxidant, and anti-inflammatory effects. These properties are largely attributed to cerium's ability to switch between the Ce^3+^ and Ce^4+^ oxidation states. In particular, this ability enables redox cycling allowing scavenging of reactive oxygen species (ROS) reducing oxidative stress.^19,20^ ROS are natural byproducts of cellular metabolism; however, excessive ROS production is a hallmark of chronic non-healing wounds. High levels of ROS can damage cellular components, perpetuate inflammation, and impair cell migration.^21,22^

To explore possible enhancement of antibacterial and antioxidant properties of cerium, PGPs and PGFs were also loaded with the natural product clove oil (clv). Clv is widely recognized for its antioxidant and antibacterial activity, largely attributed to its high eugenol content (70–90%). Eugenol disrupts bacterial cell membranes by interacting with phospholipid bilayers, increasing membrane fluidity and permeability.^24^ Studies have shown that even low concentrations of clv (0.1–1.0 mg mL^−1^) are effective against both Gram-negative and Gram-positive bacteria.^25,26^ In addition, clv is known for its potent antioxidant effects which play a crucial role in protecting cells and tissues from oxidative damage caused by ROS and free radicals.^27^

In this work, the antibacterial and antioxidant properties of PGPs and PGFs’ dissolution products have been investigated as a function of cerium and clv loading.

Antibacterial tests were performed against *Escherichia coli* (*E. coli*), a Gram-negative bacterium commonly found in wounds.^23^ Antioxidant activities were investigated *via* DPPH (2,2-diphenyl-1-picrylhydrazyl) and TPC (Total Phenolic Content) assays. Ce-free PGPs and PGFs without clv were also tested for comparison purposes. In addition, to study the physiological relevance of the PGs's antioxidant capacity, intracellular ROS levels were quantified *via* the DCFDA (2′,7′-dichlorofluorescin diacetate) assay. Cytocompatibility assays on keratinocyte cells (HaCaTs) were performed both indirectly (by using dissolution products of PGPs and PGFs) and directly, by seeding HaCaTs onto porous PGFs. Direct contact seeding of cells on coacervate-made PGFs has never been presented and demonstrates for the first time cell attachment, spreading, and proliferation on the glass surface.^28^ Wound closure after treatment with the PGPs and PGFs dissolution products was evaluated using an *in vitro* scratch assay on HaCaTs.

## 2. Experimental methods

### 2.1. Synthesis of PGPs and PGFs

**Fig. 1:**
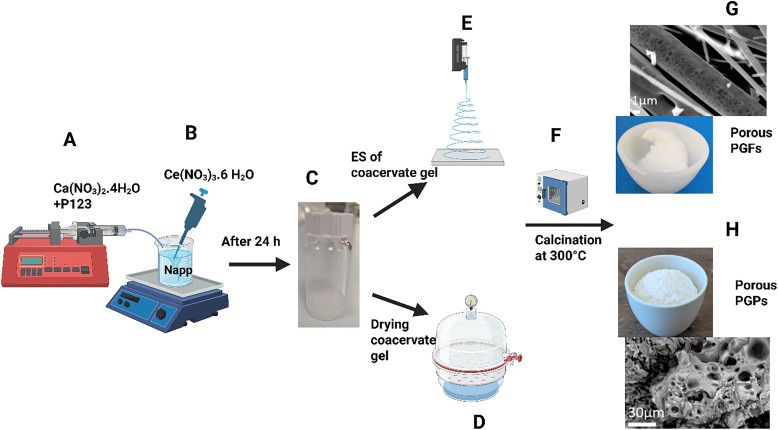
Schematic of the synthesis of PGPs and PGFs *via* coacervation: (A) addition of Ca ions and (B) of Ce ions to NaPP aqueous solution; (C) image of the coacervate gel; (D) drying of the coacervate gel in desiccator to produce PGPs; (E) ES of the coacervate gel to produce PGFs; (F) calcination of PGPs and PGFs at 300 °C; (G) SEM and optical image of PGF-unl; (H) SEM and optical image of PGP-unl.

To produce the ternary system P_2_O_5_–CaO–Na_2_O (PG-unl), 47.2 g of calcium nitrate tetrahydrate (Ca(NO_3_)_2_·4H_2_O, Fisher, >99%) was added to an aqueous solution of poly(ethylene oxide)-*block*-poly(propylene oxide)-*block*-poly(ethylene oxide) (Pluronic 123; P123, Sigma-Aldrich), prepared by dissolving 1 g of P123 in 100 mL of deionized (DI) water. The mixture was then stirred until complete dissolution of Ca(NO_3_)_2_·4H_2_O. This mixture was then added at 20 mL h^−1^ to 20 mL of a 0.16 M aqueous solution of sodium polyphosphate (Na(PO_3_)_*n*_*n* = 25, NaPP, Sigma-Aldrich, 96%) obtained by adding 40.7 g of NaPP to 100 mL of DI water, using a syringe pump (Fig. 1A).

To prepare the cerium-loaded PGPs and PGFs containing 0.1, 0.2 and 0.4 atomic% of cerium, 0.08 mL, 0.2 mL and 0.4 mL of a 2 M aqueous solution of cerium(iii) nitrate hexahydrate, (Ce(NO_3_)_3_·6H_2_O Sigma-Aldrich, 99%) were added to the mixture prepared as above, respectively (Fig. 1B). After the addition, the mixtures were stirred for one hour (h) and allowed to settle for 24 h. After the settling period, two layers were formed, a clear supernatant layer and a bottom polyphosphate coacervate gel (Fig. 1C). The aqueous layer was carefully removed, and the remaining coacervate gel was either transferred to a vacuum desiccator where it was left to dry at room temperature for 24 h to produce PGPs (Fig. 1D) or injected into a syringe at a flow rate of 2.0 mL h^−1^ and electrospun to form PGFs (Spraybase system, Kildare, Ireland) (Fig. 1E). ES is a versatile technique that involves the generation of fibres from a solution/gel upon the application of a potential difference between a nozzle and collector.^17^ A voltage range of 15–18 kV was applied across a 15 cm working distance between the nozzle and a metallic collector plate. This setup allowed successful production of cotton-like fibres. PGPs and PGFs were then calcined at 300 °C in a furnace to remove the porogen P123 (Fig. 1F). This process causes decomposition of the micelles formed by supramolecular aggregation of P123, resulting in the formation of pores in both PGPs and PGFs.

Scanning electron microscopy (SEM) and optical images of porous PGFs and PGPs are shown in Fig. 1G and H, respectively. Most of the results presented in this work refer to PGPs and PGFs calcined at 300 °C, hereafter named as PGP-unl and PGF-unl for Ce-free powders and fibres, respectively and PGP-CeX and PGF-CeX for cerium containing PGs, where X is the atomic% of cerium. When referring to uncalcined samples, they will be named PGP-unl-unc and PGF-unl-unc and PGP-CeX-unc and PGF-CeX-unc. To manufacture clv-coated PGP-CeX and PGF-CeX, 100 mg of the calcined PGPs or PGFs were immersed in a mixture of ethanol (C_2_H_6_O, Sigma-Aldrich) and clv (Sigma Aldrich) prepared by adding 81 µL of clv to ∼3 mL of ethanol, corresponding to nominal clv concentration of 3% w/v. PGPs or PGFs were soaked in this solution for 24 h, after which the mixture was centrifuged at 4000 rpm for 15 minutes (min) to precipitate the coated glass. Then, the supernatant was removed and PGPs and PGFs were left in a fume hood until all the ethanol evaporated, yielding the final PGP-clv and PGF-clv.

### 2.2. Structural characterisation

SEM images were obtained on a JSM-7100F (Jeol) at an accelerating voltage of 15 kV. Samples were mounted onto an aluminium stub using carbon conductive tape. Pores sizes were estimated using the Fiji software.

Energy dispersive X-ray spectroscopy (EDX) was performed using a WDS MagnaRay spectrometer (Hemel Hempstead, UK) to assess elemental analysis, (P, Ca, Na, Ce, O atomic%). Each element was calculated as the average of five points of the samples’ surface.

X-ray diffraction (XRD) analysis was performed using a PANalytical X’Pert spectrometer using flat plate geometry and a Ni filtered Cu-Kα radiation with a wavelength of 1.5418 Å. Data was collected using a PIXcel-1D detector with a step size of 0.0525° and a time per step of 12 s over an angular range of 2*θ* = 10–90°.

Fourier transform infrared (FT-IR) spectra were collected using a PerkinElmer spectrometer 2000-FT-IR over the range 2000–500 cm^−1^.

XPS analysis was performed on a ThermoFisher Scientific (East Grinstead, UK) Theta Probe spectrometer. XPS spectra were acquired using a monochromated Al Kα X-ray source (*hν* = 1486.6 eV) with an anode voltage of 16 kV. An X-ray spot size of ∼400 µm radius was employed in the acquisition of all spectra. Survey spectra were acquired employing a pass energy of 300 eV. High resolution, core level spectra were acquired with a pass energy of 50 eV. Quantitative surface chemical analyses were calculated from the high resolution, core level spectra following the removal of a non-linear (Shirley) background. The manufacturer's Advantage software was used which incorporates the appropriate elemental sensitivity factors and corrects for the electron energy analyser transmission function.

### 2.3. Dissolution studies

The concentration of P, Ca, Na and Ce released by PGPs and PGFs after immersion in DI water at different time points were analysed using Microwave Plasma Atomic Emission Spectroscopy (MP-AES, 4210 Agilent). In 15 mL microcentrifuge tubes, 10 mg of each sample were immersed in 10 mL of DI water and placed in an incubator-shaker at 37 °C (220 rpm) for 24, 48, and 72 h. The suspensions were centrifuged at 4500 rpm for 5 min to separate any undissolved PGPs and PGFs from the solution. Three replicates for each time point were used. Samples were filtered using a 0.45 µm filter (Millex™-GP) and diluted 1 : 50 in 2% v/v nitric acid (HNO_3_ for trace metal analysis, Fisher Chemical) for MP-AES analysis. The emission signal of the analytes was measured at 213.6, 393.4, 589.0 and 446.0 nm for P, Ca, Na and Ce, respectively. The signal of each analyte was normalized using Be, with the emission signal at 234.9 nm introduced as an internal standard at a concentration of 5 µg mL^−1^ (prepared from a 1000 µg mL^−1^ commercial stock solution obtained from PalmaCAL). The Be standard was added to all samples and calibration standards through a T-connector before nebulization. Standards of P, Ca, Na and Ce at concentrations of 0.1, 0.5, 1, 2.5, 5, 10, 25 and 50 µg mL^−1^, were freshly prepared before calibration by dilution with 2% v/v HNO_3_ from individual commercial 1000 µg mL^−1^ stock solutions (PalmaCAL). The instrumental limits of detection (LOD, based on the 3 × SD_blank_ criterion) were 0.2, 0.1, 0.1 and 0.1 µg mL^−1^ for P, Ca, Na and Ce, respectively.

Although dissolution in DI water is commonly used to assess ion release because it minimises interference, it is not physiologically relevant. Therefore, Dulbecco's phosphate-buffered saline (PBS; modified, without CaCl_2_ and MgCl_2_; Sigma-Aldrich) was also used as a more representative dissolution medium. Ion release after 3 h was measured in PBS, and the solutions were acidified, quantified, and corrected using the same protocols applied to the DI water measurements.

### 2.4. Antibacterial activity

The antibacterial properties of PGPs and PGFs were examined by assessing the effect of their dissolution products released in DI water on the growth of *E. coli* over 24 h. Colonies of bacteria grown on Tryptone Soya Agar (TSA, Oxoid) were inoculated into Tryptone Soya Broth (TSB, Oxoid) and incubated overnight at 37 °C. Subsequently, the culture was diluted to an optical density of 0.05 at 600 nm (OD_600_) in TSB and further incubated until mid-log phase (OD_600_ of 0.5–0.8). The culture was again diluted in TSB to an OD_600_ of 0.05, and the dissolution products were added in a 1 to 10 ratio. TBS inoculated with bacteria and without dissolution products (untreated) was prepared as a positive control, and pure TSB was used as the negative control. 200 µL per well of product/bacteria mix was inoculated in triplicate into a 96-well plate, which was then placed in a CLARIOstar plate reader (BMG LABTECH). The plate was incubated at 37 °C for 24 h, and the OD_600_ of each well was measured every hour, with double orbital shaking at 300 rpm for 30 seconds (s) before each cycle. The OD_600_ measurement indicates the turbidity of the solution, which correlates with the concentration of bacteria in the broth post-treatment. The obtained values were adjusted using a blank sample containing only TSB, and statistical analyses were performed using a one-way ANOVA with Dunnett's multiple comparison tests to identify significant differences between treatments.

### 2.5. Indirect cytocompatibility testing

The cytocompatibility of PGPs and PGFs after 24 h of immersion in DI water was evaluated *via* MTS (3-(4,5-dimethylthiazol-2-yl)-5-(3-carboxymethoxyphenyl)-2-(4-sulfophenyl)-2*H*-tetrazolium, Promega, G3582) and MTT (3-(4,5-dimethylthiazol-2-yl)-2,5-diphenyltetrazolium bromide) assays. These tests are commonly performed for assessing the cytocompatibility of silicates and phosphate glasses on cells.^16^

#### 2.5.1. Culture of HaCaTs

*In vitro* spontaneously transformed keratinocytes from histologically normal skin (HaCaTs; AddexBio, San Diego, USA) were used for this study. Cells between passage numbers 4 and 7 were utilized for all experiments. The cells were cultured in Dulbecco's Modified Eagle Medium (DMEM; Gibco) supplemented with 1% v/v l-glutamine (Gibco), 1% v/v Penicillin–Streptomycin (100× stock solution; final concentration: 100 U mL^−1^ penicillin and 100 µg mL^−1^ streptomycin), 10% v/v Fetal Bovine Serum (FBS; ThermoFisher), and 1 mM CaCl_2_ (prepared from 1 M stock solution). Cells were maintained at 37 °C in a humidified atmosphere containing 5% CO_2_.

#### 2.5.2. MTT assay

The metabolic activity of HaCaTs exposed to dissolution products was assessed using the MTT assay. Cells were seeded in 96-well plates at a density of 1 × 10^5^ cells mL^−1^ in the complete culture medium described above. The plates were incubated overnight at 37 °C with 5% CO_2_ to allow for cell adhesion. Following incubation, the medium was aspirated, and the adherent cells were washed twice with PBS (Gibco). A treatment medium was prepared by diluting the dissolution products in fresh culture medium at a ratio of 40 µL of dissolution products to 360 µL of medium. This treatment solution was added to the wells, and the cells were incubated for a further 24 h. Control wells received fresh medium without dissolution products.

After the 24 h exposure period, 12 µL of MTT reagent was added to each well. The plates were incubated for 3 h at 37 °C in the dark. Subsequently, the medium was aspirated, and 200 µL of dimethyl sulfoxide (DMSO, ≥99.9%; Sigma-Aldrich) was added to solubilize the formazan crystals. The plates were incubated for 30 minutes at room temperature.

Absorbance was measured at 570 nm using a microplate reader (FLUOstar Omega, BMG LabTech).

#### 2.5.3 MTS assay

In addition to the MTT assay, cytocompatibility was evaluated *via* the MTS assay. HaCaTs were seeded in 96-well plates at a density of 5 × 10^3^ cells per mL in complete culture medium and incubated overnight at 37 °C with 5% CO_2_ to allow for adhesion. After incubation, the medium was aspirated, and the adherent cells were washed twice with phosphate-buffered saline (PBS; Gibco).

A treatment medium containing the dissolution products, was prepared by diluting them in culture medium at two different volume ratios: 20 µL of dissolution products in 380 µL of medium (low concentration) and 80 µL of dissolution products in 320 µL of medium (high concentration). Control group was treated with 20 and 80 µL of PBS in the corresponding volume of medium. After incubation, 20 µL of MTS reagent was added to each well. The plates were incubated at 37 °C in the dark for 3 h. Absorbance was then measured using a microplate reader (SpectraMax iD3) at 490 nm, with a reference reading at 630 nm to correct for background signal.

### 2.6. Direct contact cytocompatibility testing

To evaluate the viability of HaCaTs when cultured in direct contact with PGFs loaded with 0.2 at% Ce, PGFs were sterilized by UV treatment for 24 h and placed in a 24-well plate. HaCaTs were harvested by trypsinization, counted, and resuspended in DMEM at a concentration of 5.8 × 10^6^ cells per mL. A volume of 103 µL of this suspension, equivalent to 6 × 10^5^ cells, was seeded directly onto each PGF sample. Cells were incubated at 37 °C with 5% CO_2_ for cell attachment for 24 h, after which fresh DMEM was added, and incubation continued for 72 h.

At the endpoint, the medium was removed, and PGFs were washed with PBS. To ensure that only cells adhered to the PGF samples were analysed, the PGFs were carefully transferred to new wells prior to staining. This step eliminated the presence of unattached cells in the original culture wells. Then, cells were incubated with Calcein-AM (Thermo Fisher Scientific, Stock: 1 mg mL^−1^, 4 µM in PBS) and Hoechst 33342 (Invitrogen, stock: 20 mM, 1 µL mL^−1^ in PBS) for 30 min at 37 °C in the dark. Following incubation, cells were washed with PBS before imaging under a fluorescence microscope (Nikon TS2 fluorescent microscope) and images were taken using (NIS Elements BR, Version 5.01).

To visualize the cytoskeleton and nuclei, first, samples were washed twice with PBS and incubated with Phalloidin-iFluor 488 Reagent (Abcam, 10 µL stock in 10 mL PBS). A volume of 500 µL was added to each well, and PGFs were incubated at room temperature for 30 min in the dark. After PBS washing, the PGFs were transferred to a separate well plate for confocal imaging to ensure that the observed cells were located directly on the fibres. Nuclear staining was then performed using DAPI (Merck, stock: 5 mg mL^−1^). A working solution was prepared by diluting 10 µL of DAPI into 490 µL PBS, and 500 µL of this solution was added to each well. Samples were incubated for 10 min at room temperature in the dark, followed by a final PBS wash. Samples were then imaged using confocal laser scanning microscopy (Nikon Ti-Eclipse A1M confocal microscope) using 20× and 40× objectives.

### 2.7. Release of clv

The release of clv was studied using UV-Visible (UV-Vis) spectroscopy as a conventional, established technique (Specord 40, Analytik Jena AG, Germany).^30,31^ Particular care was taken to avoid clv oxidation by minimizing light exposure and wrapping the containers in aluminium foil.

10 mg of PGPs and PGFs coated with clv (Sigma Aldrich) were immersed in 10 mL of PBS and placed in an orbital shaker at 90 rpm and at 37 °C. At different time points (1, 3, 6, 12, 24, 48, 72 and 168 h), 0.5 mL of the solution was taken and analysed *via* UV-vis. The solution taken for measurement was then replaced with fresh 0.5 mL of PBS. A calibration curve was generated at 280 nm using eugenol (Sigma-Aldrich) as the main component of clv. An eugenol calibration curve at 280 nm was performed by using five different eugenol concentrations (standard eugenol, Sigma Aldrich), from 0.6 to 9.7 µg mL^−1^ (*R*^2^ = 0.9995). Measurements were conducted in triplicate.

### 2.8. Antioxidant activity (DPPH and TPC assays)

The antioxidant activity of PGPs and PGFs was assessed using the DPPH and TPC assays.^30^ For the DPPH assay, first, 1 mg of clv coated PGPs and PGFs was immersed in 1 mL of methanol (CH_3_OH, Sigma-Aldrich, 99.9%, HPLC grade) and allowed to incubate overnight. Following incubation, 2.5 mL of a 0.04 mg mL^−1^ DPPH solution was added to 0.5 mL of the methanol extract containing PGPs and PGFs. The mixture, which initially appeared dark purple, gradually changed to yellow, indicating a reduction in DPPH radicals. The assay was conducted in a dark room to prevent further oxidation of the DPPH radical by light.

The absorbance of the mixture was measured at 517 nm using UV-Visible spectroscopy (Specord 40, Analytik Jena AG, Germany). A decrease in absorbance at this wavelength corresponds to the scavenging of DPPH radicals, indicating the antioxidant activity of the sample. The DPPH radical scavenging activity (DPPH RSA%) was calculated using eqn (1):1
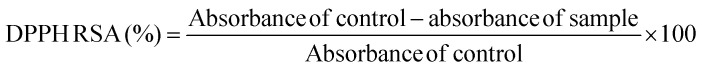


In eqn (1), the absorbance of the control represents the DPPH solution without the sample, and the absorbance of the sample corresponds to the DPPH solution mixed with the PGP or PGF dissolution product. Methanol (CH_3_OH, Sigma-Aldrich, 99.9%, HPLC grade) was used as a blank to calibrate the UV-Vis spectrometer. All experiments were performed in triplicate.

The TPC of PGPs and PGFs was determined using the Folin & Ciocalteu's phenol reagent (Sigma Aldrich).^30^ 1 mg of PGP or PGF was soaked in 1 mL of methanol for 24 h. Then, 0.5 mL of Folin & Ciocalteu's phenol reagent solution were added to the mixture. After 5 min, 2 mL of a 0.7 M sodium carbonate solution (Na_2_CO_3_, Sigma-Aldrich) (7.5 g sodium carbonate in 100 mL of DI water) was added to the mixture. After 1.5 h, the colour of mixture changed from colourless to blue. To measure the total amount of phenolic compound at 765 nm, UV-vis (Specord 40, Analytik Jena AG, Germany) was used. TPC was quantified by comparing the absorbance of the samples to a standard curve prepared using known concentrations (0.1–0.7 mg mL^−1^) of a phenolic compound (Gallic acid (C_7_H_6_O_5_ (GAE)), Sigma-Aldrich, 97.5–102.5%, by titration). Water was used as a blank and all measurements were performed in triplicate.

### 2.9. Intracellular ROS assay

ROS levels were quantified using the DCFDA/H2DCFDA - cellular ROS Assay Kit (Abcam, ab13851). HaCaTs were seeded in 96-well black-walled plates at a density of 2.5 × 10^3^ cells per well and incubated overnight to facilitate attachment. Following adhesion, the cells were washed once with PBS and subsequently labelled with the fluorogenic dye 2′,7′-dichlorofluorescin diacetate (DCFDA) in accordance with the manufacturer's protocol.

Two distinct experimental set-ups were employed to evaluate the oxidative and antioxidant properties of PGP-unl, PGP-Ce0.2-clv, PGF-unl and PGF-Ce0.2-clv.

To determine if PGs induce oxidative stress, cells were treated with PGP-unl, PGP-Ce0.2-clv, PGF-unl and PGF-Ce0.2-clv dissolution products. The treatment was prepared by adding 40 µL of the dissolution products to 360 µL of culture medium. *Tert*-Butyl hydroperoxide (TBHP) was administered at concentrations of 50 µM and 100 µM to serve as a positive control for ROS production.

To evaluate the antioxidant capacity of the PGs, cells were treated with the dissolution products (prepared as described above) in the presence of 100 µM TBHP. This setup assessed the ability of PGP-unl, PGP-Ce0.2-clv, PGF-unl and PGF-Ce0.2-clv to mitigate ROS generation induced by the oxidative stressor (TBHP).

Fluorescence intensity was measured using a fluorescence microplate reader (Spectramax iD3) with excitation and emission wavelengths set at 488 nm and 535 nm, respectively. The fluorescence signal served as a direct indicator of intracellular ROS levels.

### 2.10. *In vitro* scratch wound healing assay

To evaluate the effect of PG-unl, PG-Ce0.1, PG-Ce0.2, PG-Ce0.4, PG-unl-clv and PG-Ce0.2-clv (powders and fibres) on HaCaTs migration, an *in vitro* scratch assay was performed. Cells were seeded in 24-well plates and cultured until they reached >90% confluence.

To ensure that gap closure was attributed only to cell migration rather than proliferation, cells were pre-treated with 10 µM mitomycin C for 3 h to arrest mitosis. Following this incubation, a linear scratch was generated in the centre of each monolayer using a sterile 200 µL pipette tip. The wells were washed once with 1X PBS to remove cellular debris and replenished with fresh medium containing the dissolution products (diluted 40 µL compound in 360 µL medium).

Images of the scratch area were captured using a Nikon microscope with a 4× objective at 0, 24, and 48 h. The wound area was quantified using ImageJ software (Version 1.54p). As shown in eqn (2), the percentage of wound closure was calculated by comparing the scratch area closure at 24 and 48 h (*A*_*t*_) relative to the initial area at 0 h (*A*_0_).2
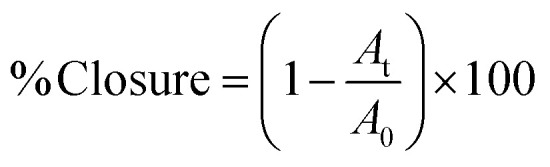


*A*_0_ is the initial wound area measured at 0 h (immediately after scratching) and *A*_*t*_ is the wound area closure measured at the specific time point (*e.g.*, 24 or 48 h).

### 2.11. Statistical analysis

For MTT, antibacterial, DPPH and TPC assay, data are presented as the mean ± standard deviation, representing at least three technical replicates. All results for MTS, ROS assays are average of 3 biological replicates, and each biological replicate is an average of three technical replicates; data are presented as mean ± standard error of the mean. For the scratch wound-healing assay, results are based on three biological replicates, with each biological replicate calculated as the mean of two technical replicates. GraphPad Prism software was used to perform all statistical analyses. Data were analysed using one-way analyses of variance (ANOVA), with Dunnett's test for MTT, MTS, antibacterial, DPPH and TPC assay and two-way analyses of variance (ANOVA), with Dunnett's test for ROS and scratch test assay.

## 3. Results

### 3.1. Structure and morphology of PGPs and PGFs

**Fig. 2:**
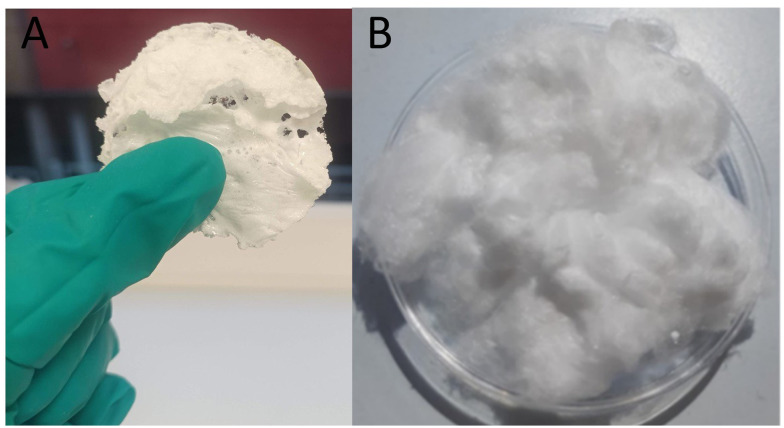
Images of (A) PGP-unl-unc and (B) PGF-unl-unc.

Representative images of PGPs and PGFs before calcination at 300 °C are shown in Fig. 2A and B, respectively. This temperature was identified as the optimal value required for producing porosity by removing the organic porogen and minimising the risk of crystallization.^15^

The XRD patterns of all PGPs and PGFs after calcination at 300 °C, show the halo at 2*θ* between 20° and 40° characteristic of the amorphous phosphate network (Fig. S1).^15^ This is in agreement with previous works on coacervate PGs powder in the P_2_O_5_–CaO–Na_2_O–Ag_2_O system, which have shown how calcination at 400 °C results in crystallisation whereas calcination at 300 °C preserves the amorphous structure.^32^

**Fig. 3:**
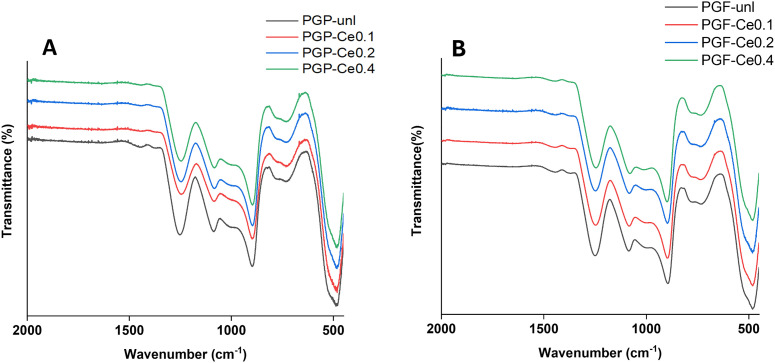
FT-IR spectra of (A) PGPs, (B) PGFs.

The structure of the phosphate network of all PGPs and PGFs, investigated *via* FT-IR spectroscopy (Fig. 3A and B, respectively), is also similar to that of PGs prepared *via* SG and MQ.^12,33^ Vibrations arising from the chain (P–O–P) groups (Q^2^) are observed at 540 cm^−1^ (bending), 745 cm^−1^ (symmetric stretching) and 900 cm^−1^ (asymmetric stretching). Stretching vibrations arising from out-of-chain groups (PO_2_)^−^ (Q^2^) are observed at 1180 cm^−1^ (symmetric) and 1250 cm^−1^ (asymmetric) and stretching vibrations related to terminal (PO_3_)^2−^ groups (Q^1^) are observed at 1000 cm^−1^ (symmetric) and 1150 cm^−1^ (asymmetric). Structure does not change significantly with composition.

**Fig. 4:**
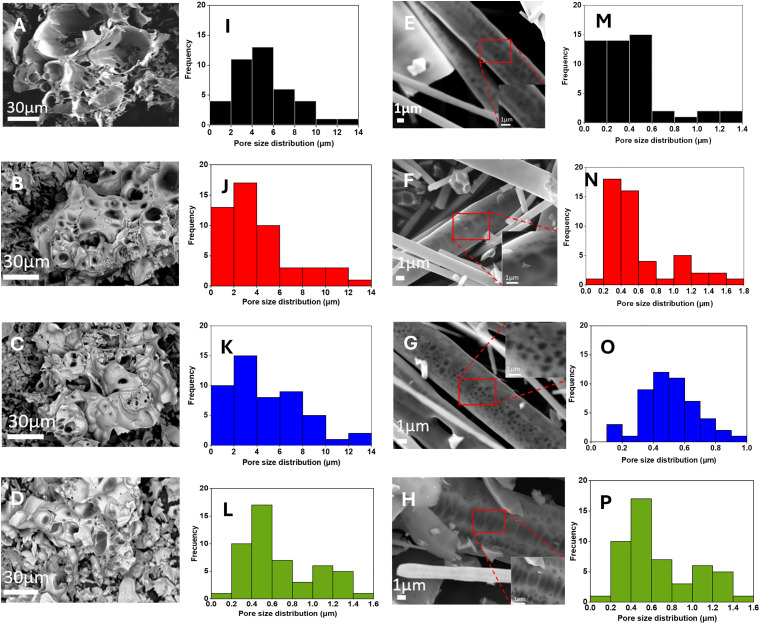
SEM images of porous PGPs (A–D) and porous PGFs (E–H); pore size distributions of porous PGPs (I–L) and porous PGFs (M–P). PGP-unl (A and I); PGP-Ce0.1 (B and J); PGP-Ce0.2 (C and K) and PGP-Ce0.4 (D and L); PGF-unl (E and M); PGF-Ce0.1 (F and N); PGF-Ce0.2 (G and O) and PGF-Ce0.4 (H and P). Pore size distributions were determined using the Fiji software.^34^

Morphology of all samples was investigated *via* SEM. Representative SEM images of calcined PGPs and PGFs are shown in Fig. 4A–D and E–H, respectively.

All SEM images show extended porosity; pore size distributions of all samples have been identified and presented as bar charts in Fig. 4I to L (PGPs) and Fig. 4M to P (PGFs), respectively.

In the SEM images of PGPs (Fig. 4A–D), the porosity spans across a size range between 1 and 14 µm (macropores) with most pores in the size range of 2–10 µm (Fig. 4I–L). Some variations in distribution can be observed between samples. PGP-unl (Fig. 4I) shows the widest range of pores, most are 4–6 µm in size, but some reach up to 12–14 µm. PGP-Ce0.1 (Fig. 3J) exhibits a higher percentage of smaller pores (<4 µm) compared to the other samples, which show a more uniform distribution across larger pore sizes. In PGP-Ce0.2 most pores are about 2–4 µm, with a spread from roughly 2 µm up to 10 µm and very few pores in the range 10–14 µm. Pores distribution of PGP-Ce0.4 (Fig. 4L) shows pore sizes mostly between 2 and 6 µm, with a small number of larger pores up to about 10–12 µm.

In the SEM images of PGFs (Fig. 4E–H), extended porosity is also observed. However, pore sizes are significantly smaller than those observed in PGPs. Most pores falling within the range ∼0.2–1.4 µm (Fig. 4M–P). Fig. 4M shows that in PGF-unl most pores are very small, around 0.2–0.4 µm, with numbers of pores dropping significantly for sizes over 0.6 µm and few pores are observed between 0.6–1.4 µm. In PGF-Ce0.1 and PGF-Ce0.4 (Fig. 4N and P) pores mostly are between 0.2–0.8 µm, while in PGF-Ce0.2 (Fig. 4O), the pores exhibit a broader distribution of pore sizes.

On the contrary, SEM images of calcined PGP-unl (Fig. S2A) and PGF-unl (Fig. S2B) obtained without addition of the porogen P123 show smooth surfaces and absence of porosity, as expected.

### 3.2. Composition and oxidation state of cerium

Elemental composition (atomic%) of all calcined PGPs and PGFs was assessed *via* EDX (Table 1). P, Ca and Na content were chosen on the basis of previous investigation on SG, MQ and coacervate PGs that showed good biocompatibility.^35^ The O atomic percentage varies between 53.4% and 63.5%, while Na and P concentrations range from 4.1% to 5.3% and 22.1% to 28.0%, respectively. Ca is present at levels between 9.1% and 14.1%.

**Table 1:** Elemental composition (atomic%) of PGPs and PGFs measured *via* EDX. Values represent mean ± standard deviation from three measurements per sample (*n* = 3)

Sample name	Element (atomic%)				
	O	Na	P	Ca	Ce
**PGP-un**l	59.4 ± 0.4	4.5 ± 0.1	24.8 ± 0.2	11.3 ± 0.2	—
**PGP-Ce0.1**	58.8 ± 0.8	5.2 ± 0.6	25.1 ± 0.7	10.8 ± 1.0	0.1 ± 0.1
**PGP-Ce0.2**	60.2 ± 0.5	5.1 ± 0.8	23.7 ± 0.4	10.8 ± 0.4	0.2 ± 0.1
**PGP-Ce0.4**	63.5 ± 0.4	4.9 ± 0.3	22.1 ± 0.2	9.1 ± 0.1	0.4 ± 0.1
**PGF-un**l	60.7 ± 0.4	4.1 ± 0.1	25.1 ± 0.2	10.1 ± 0.2	—
**PGF-Ce0.1**	53.4 ± 0.4	4.3 ± 0.1	28.0 ± 0.2	14.1 ± 0.1	0.1 ± 0.1
**PGF-Ce0.2**	60.9 ± 0.4	5.3 ± 0.3	23.5 ± 0.2	10.1 ± 0.1	0.2 ± 0.1
**PGF-Ce0.4**	57.8 ± 0.5	4.6 ± 0.2	25.7 ± 0.3	11.6 ± 0.2	0.3 ± 0.1

EDX can only give elemental information. However, it is well known that the oxidation state of the therapeutic ion cerium, (3+ or 4+) plays an important role in biological systems.^36^ Therefore, XPS analysis was performed to identify the oxidation state of cerium. Spectra of PGFs with Ce contents lower than 0.4 atomic%, have a very poor signal-to-noise due to small surface areas and trace Ce levels. Therefore, peak fitting was performed exclusively for the PGF-Ce0.4 sample, which contains the highest concentration of Ce (Fig. S3).

**Fig. 5:**
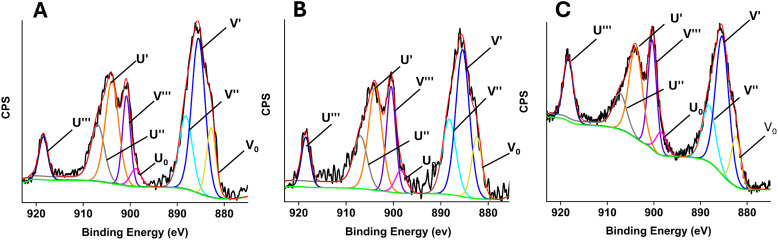
Fitted Ce 3d_3/2_ and Ce 3d_5/2_ XPS spectra of Ce^3+^ and Ce^4+^ for (A) PGP-Ce0.1, (B) PGP-Ce0.2 and (C) PGP-Ce0.4.

XPS spectra of porous PGP-Ce0.1, PGP-Ce0.2 and PGP-Ce0.4 are shown in Fig. 5A, B and C, respectively.

XPS spectra of the Ce 3d region (Fig. 5) indicate the presence of both Ce^3+^ and Ce^4+^. The Ce 3d spectra are complex due the satellite structure associated with the final state occupation of the Ce 4f level.^37^ The U and V nomenclature for peak assignment is based on that employed by Burroughs *et al.*^38^ The spectra display the characteristic spin–orbit split components corresponding to Ce 3d_5/2_ (V peaks) and Ce 3d_3/2_ (U peaks). For Ce^4+^, the characteristic peaks corresponding to the 3d_5/2_ state occur at binding energies of 888.2 eV (V′′) and 900.8 eV (V‴) and those corresponding to the 3d_3/2_ state occur at 907.1 eV (U″) and 918.5 eV (U‴). In contrast, the peaks associated with Ce^3+^ corresponding to the 3d_5/2_ are observed at binding energies of 882.7 eV (V_0_) and 885.5 eV (V′), those corresponding to the 3d_3/2_ are observed at 898.9 eV (U_0_) and 904.1 eV (U′).

The relative intensity of the Ce^3+^ and Ce^4+^ peaks, quantified by integrating the areas under the respective peaks, indicate that PGP-Ce0.1, PGP-Ce0.2, PGP-Ce0.4 and PGF-Ce0.4 contain ∼39–40% of Ce^3+^ and ∼60–61% of Ce^4+^ (Table 2) with a Ce^3+^/Ce^4+^ of ∼0.63, regardless of Ce content.

**Table 2:** Concentration of Ce 3d _3/2_ and 3d_5/2_ derived from fitting Ce 3d_3/2_ and Ce 3d_5/2_ XPS spectra of Ce^3+^ and Ce^4+^ for PGPs and PGFs

Sample	Ce^3+^ 3d_3_/_2_ (%)	Ce^3+^ 3d_5_/_2_ (%)	Ce^3+^ total (%)	Ce^4+^ 3d_3_/_2_ (%)	Ce^4+^ 3d_5_/_2_ (%)	Ce^4+^ total (%)	Ce^3+^/Ce^4+^
PGP-Ce0.1	12.7	26.3	39.0	24.7	36.3	61.0	0.63
PGP-Ce0.2	13.4	25.5	38.9	24.5	36.7	61.2	0.63
PGP-Ce0.4	14.3	25.7	40.1	23.5	36.4	59.9	0.66
PGF-Ce0.4	11.5	27.1	38.6	34	27.3	61.3	0.63

Knowledge of the Ce^3+^ and Ce^4+^ content, has allowed the formulation of the composition of PGPs and PGFs in terms of oxide mol%, considering both CeO_2_ and Ce_2_O_3_ (Table S1).

The P_2_O_5_ content ranges from 48.6% to 50.8%, while CaO varies between 39.5% and 43.6%. According to the literature, PGs exhibit excellent biocompatibility when P_2_O_5_ is within 45–50 mol%.^39^

### 3.3. Ion release studies

**Fig. 6:**
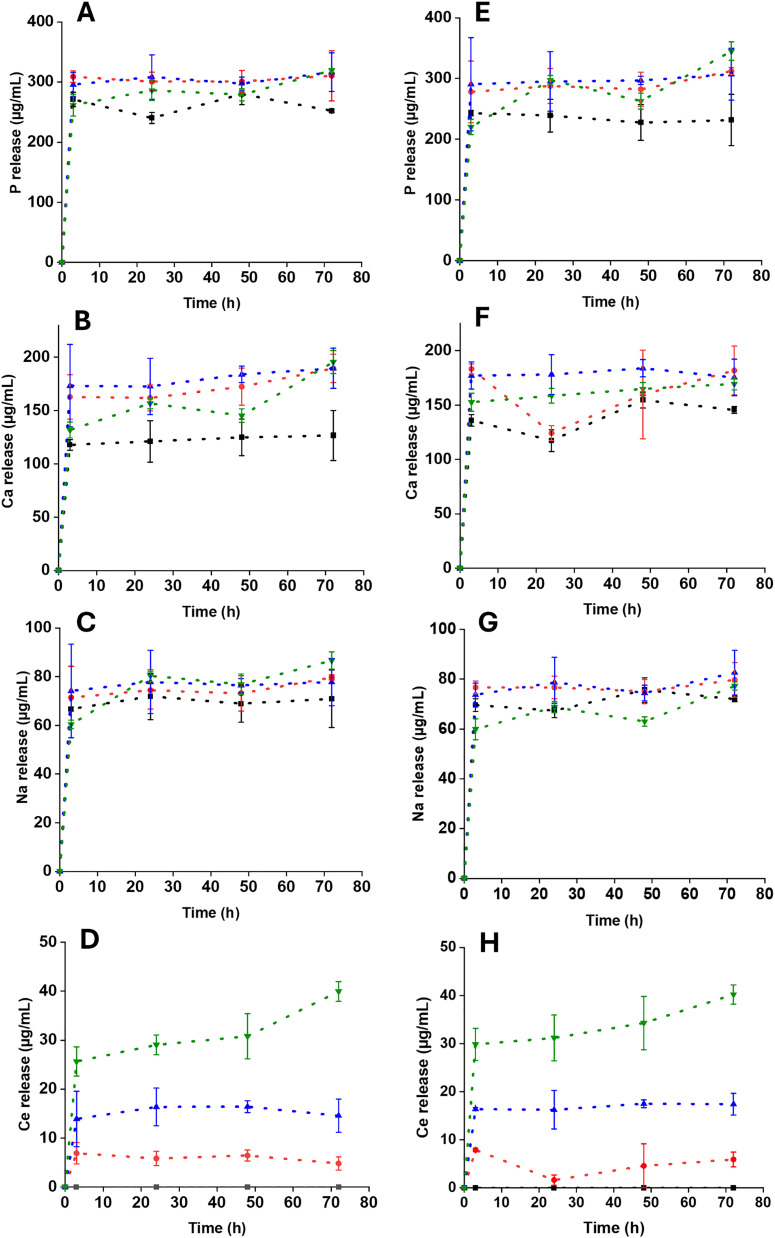
Release of P, Ca, Na and Ce from PGPs (A–D) and PGFs (E–H) after immersion in DI water up to 72 h. Error bars indicate the mean ± standard deviation (*n* = 3).

In order to assess the release behavior of PGPs and PGFs over time, the concentrations of P, Ca, Na and Ce were assessed after 3, 24, 48 and 72 h of immersion in DI water. Release profiles are shown in Fig. 6(A–D) for PGPs and Fig. 6(E–H) for PGFs respectively.

Release trends of all ions are similar for PGPs and PGFs with most of the release occurring in the first 3 h. Release of P and Ca from PGPs and PGFs is the lowest for the Ce-free samples and increases with the Ce content. For example, after 24 h of immersion, PGP-unl releases 238 µg mL^−1^ of P and 121 µg mL^−1^ of Ca whereas PGP-Ce0.4 releases 297 µg mL^−1^ of P and 156 µg mL^−1^ of Ca; similarly, PGF-unl releases 240 µg mL^−1^ of P and 121 µg mL^−1^ of Ca whereas PGF-Ce0.4 releases 280 µg mL^−1^ of P and 160 µg mL^−1^ of Ca.

The release of Na is less dependent on the Ce content, and it is similar for all compositions. After 24 h, PGP-unl and PGP-Ce0.4 release 71 µg mL^−1^ of Na and 70 µg mL^−1^ of Na, respectively; PGF-unl and PGF-Ce0.4 release 67 µg mL^−1^ of Na and 71 µg mL^−1^ of Na, respectively. The trend in the release of Ce is similar for PGPs and PGFs. Ce release increases with increasing the Ce loading, with PGP-Ce0.4 and PGF-Ce0.4 showing the highest release, 39 µg mL^−1^ and 40 µg mL^−1^, respectively.

An ion release study was also conducted in PBS to evaluate the release behaviour of P, Ca and Ce in a buffered medium that more closely simulates the *in vivo* environment. Release of Na could not be measured due to the high Na content in PBS. In Fig. S4, release of P, Ca and Ce after 3 h of immersion of PGPs (Fig. S4A–C) and PGFs (S4D–F) in PBS and DI water are presented.

Interestingly, a higher release of P and Ca is observed in PGs immersed in PBS compared to DI water. Ce release is overall much lower (<30 µmL^−1^) than that of P and Ca and differences between release in PBS and DI water are not significant for PGPs. Release of Ce from PGFs in PBS follow an unusual path.

Degradation in PGs takes place primarily through the ionic exchange of soluble ions, which is strongly dependent on the surrounding media, in particular on the ionic strength.^40,41^ Therefore, a detailed investigation, out of the scope of this work, would be needed to better understand how the kinetic of dissolution related to the ionic strength of the medium.

### 3.4. Antibacterial activity

**Fig. 7:**
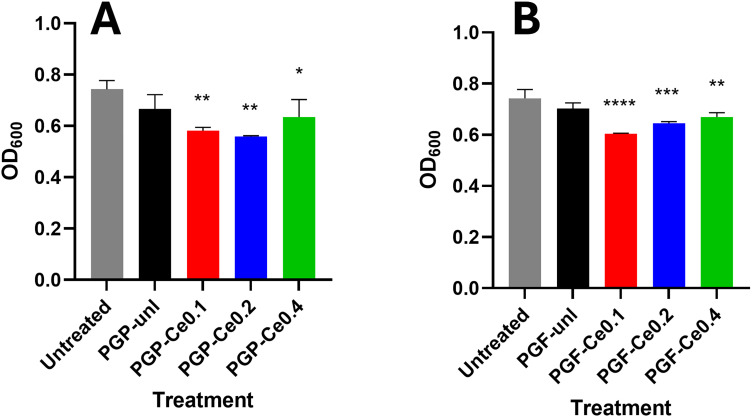
Antibacterial activity of (A) PGPs and (B) PGFs against *E. coli*. of dissolution products (obtained after 24 h of immersion of PGPs and PGFs in DI water). Error bars represent the standard deviation over technical replicates (*n* = 3). Statistical significance was determined using one-way analysis of variance (ANOVA) followed by Dunnett's *post hoc* test; *p*-values: *p < 0.05; **p < 0.01; ***p < 0.001; ****p < 0.0001.

The antibacterial effects of the dissolution products (obtained after 24 h of immersion in DI water) against *E. coli* are shown in Fig. 7A for PGP-unl and PGP-CeX and in Fig. 7B for PGF-unl and PGF-CeX (24 h incubation time).

Compared to the untreated control, the unloaded powder (PGP-unl) and fibre (PGF-unl) samples showed no significant antibacterial effect. In contrast, cerium-containing PGs demonstrated antibacterial activity, with PG-Ce0.1 and PG-Ce0.2 (in both powder and fibre forms) showing the strongest effects. The antibacterial activity of cerium is due to the disruption of the bacterial cell wall and membrane, interfering with essential metabolic functions like respiration. However, additional antibacterial effects due to pH changes cannot be excluded, given that the PGs-Ce0.4 shows a slightly lower activity than PGs-Ce0.2.^42^

### 3.5. Cytocompatibility on HaCaTs: indirect and direct testing

Indirect cytocompatibility testing of different concentrations of PGPs and PGFs dissolution products (20, 40 and 80 µL in 360 µL of medium) on HaCaTs was investigated.

**Fig. 8:**
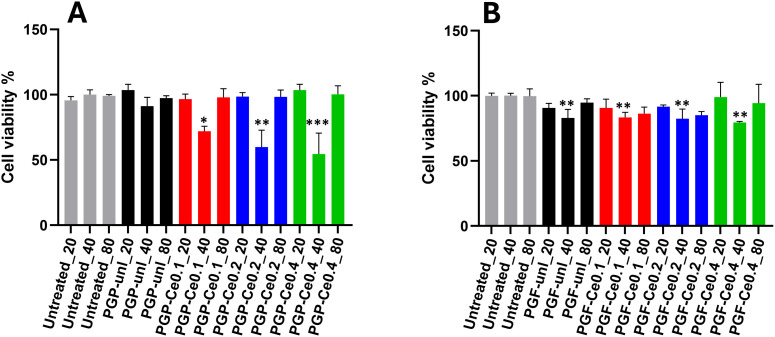
Viability of HaCaTs when exposed to (A) PGP's and (B) PGF's dissolution products (24 h immersion) for three ratios dissolution products/medium (20, 40 and 80 µL in, 380, 360 and 320 µL of medium). Data for 40 µL are mean ± standard deviation (*n* = 3 technical replicates; one-way ANOVA with Dunnett's test). Data for 20 and 80 µL are mean ± standard error of the mean (*n* = 3 biological replicates; each averaged from three technical replicates; two-way ANOVA with Dunnett's multiple comparisons). *p*-Values: *p < 0.05; **p < 0.01; ***p < 0.001.

As shown in Fig. 8, HaCaTs viability remained high (>70%) and comparable to the positive control (untreated) for most of the treatment groups. Materials with such viability values are considered non cytotoxic^43^ Only PGP-Ce0.2 and PGP-Ce0.4 at 40 µL dilution show lower cell viability values (60% and 54%, respectively).

Whilst indirect cytocompatibility testing of PGs are commonly presented, direct seeding of cells on PGs is much more challenging, with only few examples presented on PGs prepared *via* SG^12^ and MQ.^44^ To the best of our knowledge, no previous example of direct seeding on PGs prepared *via* coacervation has been presented.

Here a representative sample (PGF-Ce0.2) was selected for exploring direct seeding of HaCaTs.

**Fig. 9:**
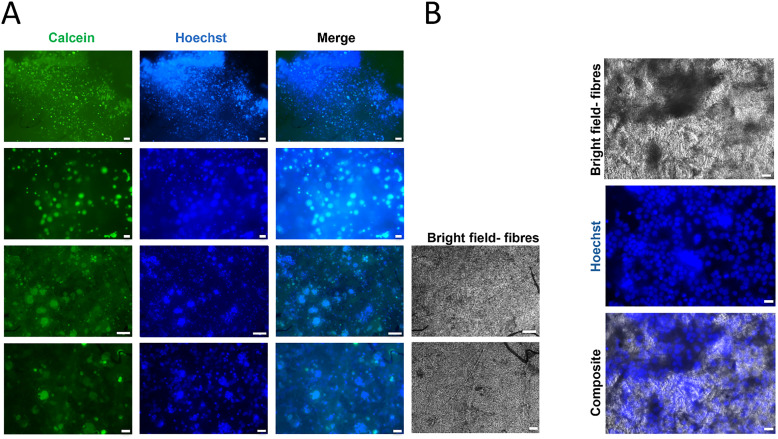
Cytocompatibility of PGF-Ce0.2 assessed *via* fluorescence microscopy. (A) Cells cultured on PGFs stained with Calcein-AM and Hoechst and imaged with a Nikon TS2 fluorescent microscope using 4×, 10× and 20× objectives, (B) bright field images of the field of views showing PGFs networks. Images are representative of 3 biological replicates. Scale bars: 100 µm.

After 72 h of culturing in 48-well plates., live cell staining with Calcein-AM dye, a common indicator of cell viability (green fluorescence) was performed to assess whether the cells were viable in direct contact with the sample.^45^ Simultaneously, cell nuclei were labelled with Hoechst dye (blue fluorescence) for nuclear staining of live cells. Fluorescence images (Fig. 9A) clearly show densely packed PGF-Ce0.2 fibres with HaCaTs clusters emitting green fluorescence (Calcein-AM) and with blue stained nuclei (Hoechst), indicating presence of viable cells in the PGF networks. The merged images clearly demonstrate colocalization of viable cells with nuclei within the PGF scaffolds (Fig. 9A). Bright field images were also acquired, along with fluorescent images, to identify PGFs networks, further supporting colocalization of viable cells with PGFs (Fig. 9B). The first two bright field images are placed next to the fluorescence images from Fig. 9A for direct comparison at the same magnification. The other three images are displayed separately to provide higher-magnification views, emphasizing cell nuclei distribution and their alignment within the PGFs structure.

**Fig. 10:**
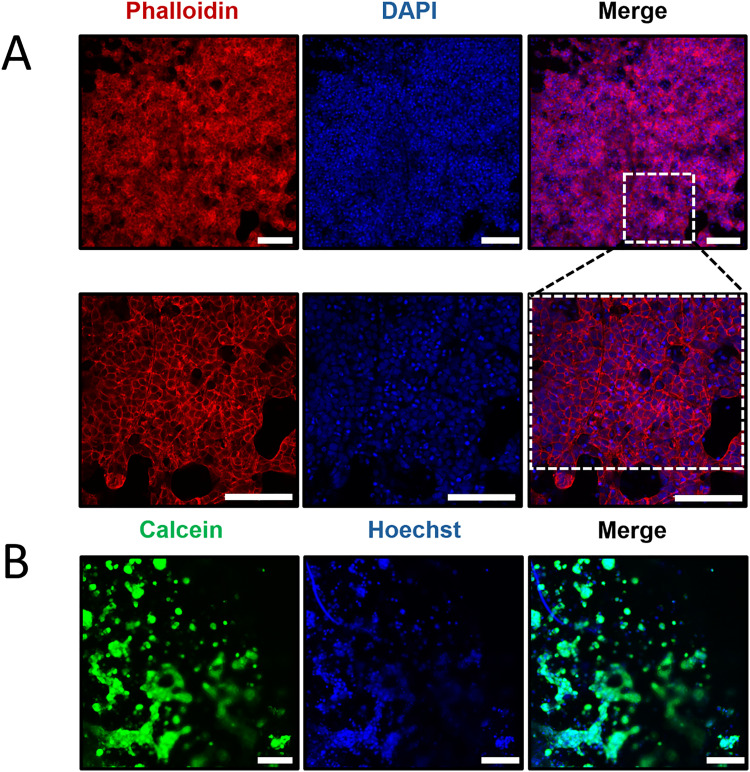
Confocal microscopy to determine structural integrity of HaCaTs stained with (A) phalloidin and DAPI and (B) Calcein-AM and Hoechst cultured on PGF-Ce0.2. Images were acquired with a Nikon Ti-Eclipse A1M confocal microscope using 20× and 40× objectives. Images are representative of 3 biological replicates. Scale bars: 100 µm.

To further evaluate cytocompatibility, HaCaTs seeded on PGF-Ce0.2 were fixed with 1% paraformaldehyde, stained for F-actin (phalloidin, red), nuclei (DAPI and Hoechst, blue) and live cells (Calcein-AM, green), and then imaged at high resolution by confocal microscopy (Fig. 10). Fig. 10A shows densely packed cell clusters on the fibrous network of PGF-Ce0.2 stained in red (phalloidin, F-actin) and blue (DAPI, nuclei) at two different magnifications (20× and 40×), along with the merged images. While the F-actin staining in red demonstrated structurally intact cells with physiological morphology, the nuclear staining evidenced homogenously packed and distributed cells (Fig. 10A). In addition, Fig. 10B presents images of HaCaTs cultured on PGF-Ce0.2 and stained with Calcein-AM and Hoechst at 20× magnification, where Calcein-AM identifies live cells and Hoechst stains nuclei, together providing complementary information on cell viability, attachment and distribution. These results demonstrate that the fibrous network of PGF-Ce 0.2 provides a biocompatible support that preserves the structural integrity and survival of HaCaTs, making it a promising candidate for skin tissue engineering.

### 3.6. PGPs and PGFs coated with clv: release, antioxidant and antibacterial properties

Clv was selected for its strong antioxidant capacity, which helps reduce oxidative stress and supports tissue repair, making it valuable for wound healing applications. Two samples, PGP-Ce0.2 and PGF-Ce0.2, were coated with clv due to their strong biological performance, particularly their marked antibacterial activity. PGP-unl and PGF-unl were also coated for reference purposes.

**Fig. 11:**
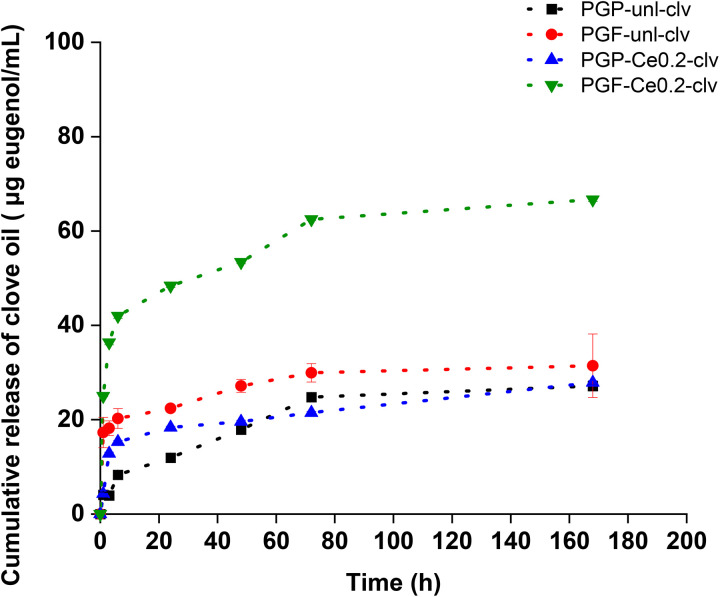
(A) Cumulative release profile of clv from PGP-unl-clv, PGF-unl-clv, PGP-Ce0.2- clv and PGF-Ce0.2-clv in an PBS at 37 °C. Values are given as mean ± standard deviation (*n* = 3).

Release of clv over time and the antioxidant properties of all four samples were investigated. The cumulative release profile of clv over 7 days exhibit a significant initial release within the first 6 h, followed by a slower and more sustained release phase (Fig. 11). These results agree with our previous work on PGFs loaded with gallium (Ga) and clv.^30^ This could be attributed to the release of clv molecules that were initially physically adsorbed on the outer surface of porous PGPs and PGFs during the first 6 h, followed by a slower release of molecules trapped within the pores or those interacting with Ga or Ce.^30,46^ It is interesting to note that PGF-unl-clv and PGF-Ce0.2-clv show higher release of clv compared to PGP-unl-clv and PGP-Ce0.2-clv. This could be due to the fibrous morphology of PGFs, which allows for a higher adsorption of clv. Consequently, in PGFs, the presence of cerium affects clv release as PGF-Ce0.2-clv exhibits a higher release profile compared to PGF-unl-clv. This is also true for PGP-Ce0.2-clv up to 7 days.

**Fig. 12:**
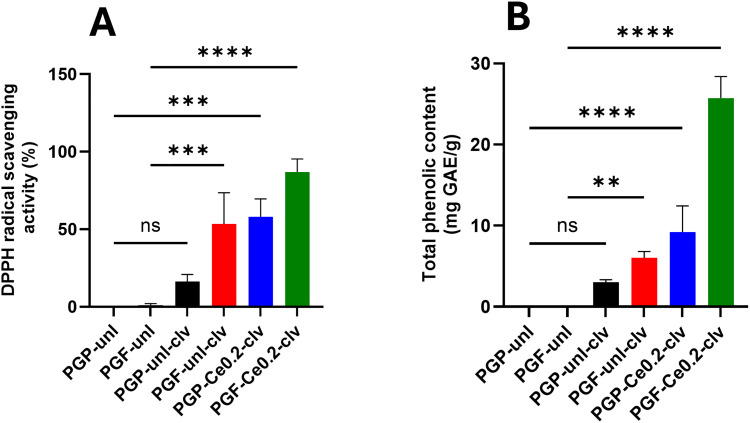
(A) DPPH radical scavenging activity and (B) TPC assay for PGP-unl, PGF-und, PGP-unl-clv, PGF-unl-clv, PGP-Ce0.2-clv, PGF-Ce0.2-clv, Values are given as mean ± standard deviation (*n* = 3). Data were analysed using one-way ANOVA (Dunnett test). *p*-Values: *p < 0.05; **p < 0.01; ***p < 0.001; ****p < 0.0001.

The antioxidant properties of PGP-unl-clv, PGP-Ce0.2-clv, PGF-unl-clv, and PGF-Ce0.2-clv were also investigated *via* DPPH and TPC assay (Fig. 12A and B, respectively). For each measurement, the effect of clv addition and the synergistic effect of clv and Ce ions on the antioxidant properties were studied. As expected, both PGP-und and PGF-und exhibited no antioxidant activity, with DPPH scavenging and TPC values being both 0, consistent with the absence of clv or Ce. The incorporation of Ce and clv imparts antioxidant properties to the glasses. PGP-unl-clv and PGF-unl-clv have a DPPH value of 16% and 50%, respectively and TPC values of 3 mg GAE g^−1^ and 5 mg GAE g^−1^, respectively. This difference is likely due to the fibrous morphology of the PGFs. Upon the addition of Ce to PGP-unl-clv and PGF-unl-clv, the DPPH increases from 16% to 53% and from 50% to 87%, respectively and TPC values increase from 3 to 9 mg GAE g^−1^ and from 5 to 25 mg GAE g^−1^, respectively.

**Fig. 13:**
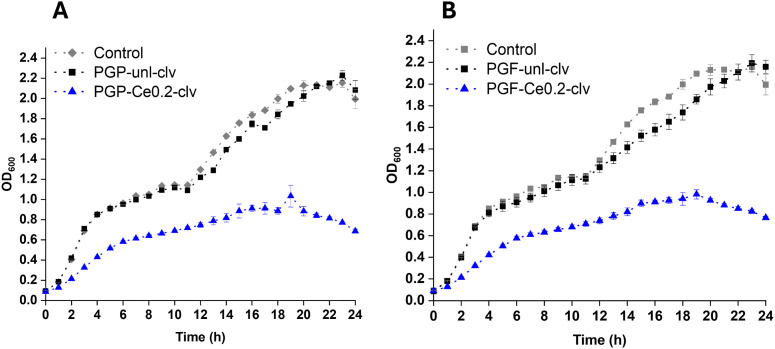
Antibacterial activity of (A) PGP-clv and (B) PGF-clv against *E. coli*. All tests have been performed in PGPs and PGFs dissolution products after 24 h dissolution in DI water. Error bars represent the standard deviation over technical replicates (*n* = 3). Data were analysed using one-way ANOVA (Dunnett test).

In addition to the antioxidant properties, the antibacterial effect of PGP-unl-clv, PGP-Ce0.2-clv (Fig. 13A) and PGF-unl-clv and PGF-Ce-0.2-clv against *E. coli* were also studied (Fig. 13B). Statistically significant differences (*p* < 0.0001) were observed between PGP-Ce0.2-clv and PGF-Ce0.2-clv compared with the control. The results suggest that the combined effect of the different components of the modified glasses against the *E. coli*, overwhelms the bacterial defences. Clv disrupts the bacterial membrane and increases its permeability,^24^ while the Ce^3+^/Ce^4+^ redox cycle produces ROS that disrupt key intracellular enzymes.^47^ These findings align with previous work that showed how mesoporous bioactive glass nanoparticles loaded with 1.5–3% (w/v) of clv exhibited significant antibacterial activity against *E. coli*.^46^

### 3.7. Evaluation of oxidative stress and antioxidant potential

**Fig. 14:**
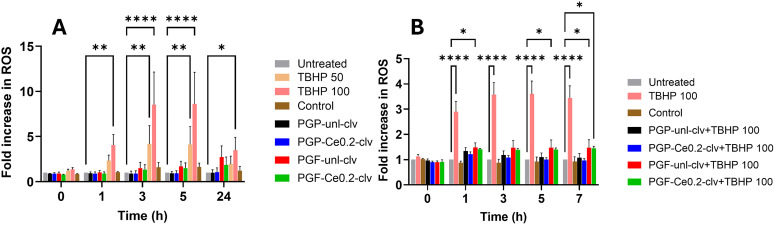
Intracellular ROS response in HaCaTs over time. (A) ROS levels following treatment with dissolution products alone, (B) antioxidant response under TBHP-induced oxidative conditions. Data are presented as mean ± standard error of the mean (*n* = 3 biological replicates), where each biological replicate represents the average of three technical replicates. Statistical analysis was performed using two-way ANOVA (Dannett's multiple comparison test). A *p*-value < 0.05 was considered statistically significant; *p*-values: *p < 0.05; **p < 0.01; ***p < 0.001, ***p < 0.0001.

The ability of PGP-unl-clv, PGP-Ce0.2-clv, PGF-unl-clv and PGF-Ce0.2-clv to modulate intracellular oxidative stress was investigated using the DCFDA fluorescence assay. This assessment had two objectives: first, to determine if the PGs themselves induce ROS generation (Fig. 14A), and second, to evaluate their antioxidant capacity against an oxidative stressor (Fig. 14B).

The TBHP produced a marked increase in fluorescence intensity, confirming strong intracellular ROS generation. In contrast, treatment with dissolution products from PGP-unl-clv and PGP-Ce0.2-clv resulted in fluorescence levels close to the control at 24 h, indicating no meaningful intrinsic ROS induction. PGF-unl-clv and PGF-Ce0.2-clv showed a small increase in fluorescence relative to the control; however, this response remained substantially lower than TBHP at both 50 and 100 concentrations. Overall, these results indicate that the dissolution products do not strongly induce oxidative stress.

To study the antioxidant scavenging activity, cells were treated with the dissolution products and TBHP (Fig. 14B). While TBHP treatment alone resulted in a high fluorescence signal indicative of strong oxidative stress, the addition of compounds PGP-unl-clv, PGP-Ce0.2-clv, PGF-unl-clv and PGF-Ce0.2-clv significantly reduced this response. The fluorescence intensity in the treated PGs was markedly lower than that of the TBHP-only group, suggesting that these PGs possess strong antioxidant properties and are capable of scavenging ROS or protecting cells from oxidative damage.

### 3.8. Scratch test for evaluation of wound closure

**Fig. 15:**
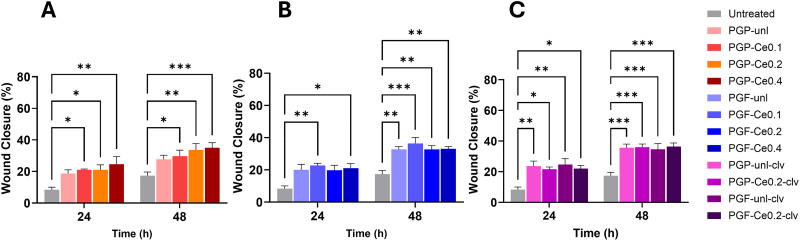
*In vitro* scratch wound healing assay using HaCaTs. Quantification of wound closure (%) at 0, 24, and 48 h, demonstrating the effect of dissolution products of (A) PGPs, (B) PGFs, and (C) PGP-clv and PGF-clv on cell migration and wound closure. Data are presented as mean ± standard error of the mean (*n* = 3 biological replicates), where each biological replicate represents the average of two technical replicates. Statistical analysis was performed using two-way ANOVA (Dannett's multiple comparison test). A *p*-value < 0.05 was considered statistically significant; *p*-values: *p < 0.05; **p < 0.01; ***p < 0.001.

The effect of dissolution products of PGPs and PGFs into wound healing was evaluated using an *in vitro* scratch assay on HaCaTs. Quantitative analysis of the wound area at 24, and 48 h revealed that treatment with dissolution products of PGPs and PGFs unloaded and loaded with Ce and clv, significantly promoted wound closure compared to the untreated control (Fig. 15 and Fig. S5). For PGPs, wound closure increased over time for all groups. At 24 h, untreated cells showed low closure (∼ 8%), while PGP-unl and Ce-loaded PGPs reached approximately 18–25%. The highest value at this time point was observed for PGP-Ce0.4 (∼24%). At 48 h, closure further increased, with PGP-Ce0.4 reaching the highest percentage (∼35%), followed by PGP-Ce0.2 (∼34%). All Ce-containing groups showed significantly greater closure than untreated control, particularly at 48 h.

For PGFs, a similar time-dependent increase was observed. At 24 h, closure ranged ∼18–22% for PGF-Ce compared to ∼10% in untreated cells. By 48 h, PGF-Ce0.2 showed the highest wound closure (∼35%). These values were significantly higher than the untreated group (∼18%), indicating enhanced cell migration with cerium incorporation. In PGs loaded with clv, wound closure was further improved. Notably, incorporation of clv further increased wound closure as early as 24 h, and this improvement remained significant at 48 h. At 24 h, clv-containing PGs showed closure of approximately 20–25%, compared to ∼10% in untreated cells. The most significant effects were observed at 48 h, where all PGs achieved the closure ∼34–37%. These values represent the greatest wound healing closure% among all tested conditions. Interestingly, the combination of Ce and clv provided the highest wound closure values at 48 h, indicating an enhanced pro-migratory effect compared with the corresponding unloaded PGs.

## 4. Discussion

In this study, we have demonstrated the feasibility of the coacervation method combined with supramolecular templating to produce porous PGPs and PGFs loaded with various amounts of cerium ions (0.1, 0.2 and 0.4 at%) and 3 w/v% of clv. The primary aim was to achieve hierarchical porosity along with multifunctional bioactivity (antibacterial and antioxidant activity), while preserving cytocompatibility towards keratinocytes. The use of P123 as a porogen successfully generated macropores (1–14 µm) in PGPs and meso-/macropores (0.2–4 µm) in PGFs upon calcination at 300 °C. P123 typically aids in forming mesopores (2–50 nm in diameter) by creating a regular network of pores as it self-assembles into micelles.^48,49^ However, SEM images showed a range of much larger pores of irregular shape, suggesting that the synthesis conditions may have caused some aggregation of P123 micelles. The presence of macropores is nevertheless very beneficial; macropores can facilitate cell differentiation and tissue formation^4^ and allow cells to infiltrate and move through the material, which is vital for tissue regeneration. The size of the macropores can also influence stem cell differentiation. For example, pores larger than 250 µm support osteogenic differentiation and vascularization, while pores smaller than 125 µm support stemness and prevent differentiation.^4^ EDX results confirmed that all formulations remained within the biocompatible compositional window (P_2_O_5_: 46–51 mol%, CaO: 39–43 mol%).

Indirect cytocompatibility testing showed cell viability above 70% for all PGFs, as well as for PGP-unl and PGP-Ce0.1, except for PGP-Ce0.2 and PGP-Ce0.4. Slightly differences in cytocompatibility could be due in difference in pH or polyanionic structure.

Overall, these findings indicate that the released ions are largely non-cytotoxic.^50^ This is in agreement with previous work that showed how PGFs in the system 18MgO–10CaO–24Na_2_O–45P_2_O_5_–3CeO_2_ (mol%) prepared *via* melt-spinning process were no toxic to HaCaTs after 7 days of indirect exposure.^51^

Direct biocompatibility testing gave remarkable results, being the first on this kind of coacervate-made PGs, and demonstrated extensive HaCaTs attachment, spreading, adherence, cytoskeletal integrity, and cell viability. Although direct cell seeding on coacervate-derived PGFs has not yet been explored, similar fibrous scaffolds based on silicate glasses (doped with 5 wt% cerium) demonstrated excellent cytocompatibility. In those studies, osteoblast cells (MC3T3-E1) were observed to adhere and spread effectively along the fibrous network.^29^

Dissolution profiles of P, Ca, Na, and Ce in DI water showed mainly release within the first 3 h, plateauing by 24 h, a pattern beneficial for an initial therapeutic release followed by sustained activity. Increase in Ce loading, slightly enhances P and Ca release, possibly due to network modifications (increased of number of non-bridging oxygens, NBOs) weakening the phosphate network. However, in previous studies on Ga-doped PGFs,^30^ Ga incorporation was found to reduce phosphate release, possibly due to a cross-linking effect.^51^ Cerium release increased proportionally with increasing concentration. Dissolution products of PGP-CeX and PGF-CeX exhibited great inhibition of *E. coli* growth than the control. These results are consistent with previous findings demonstrating Ce antibacterial activity. Lapa *et al.* reported a Ce ion concentration lower than 10 µg mL^−1^ released into the bacterial growth medium from Ce-doped PGPs *via* MQ showed inhibition of *E. coli* growth over 24 h.^19^ CeO_2_ loaded bioglass 45S5 (CeO_2_ : 0.25, 0.5, 1, and 2 mol%) prepared *via* MQ were also shown to have antibacterial activity against *E. coli*, increasing with cerium content.^21^

However, additional effect could contribute such as pH changes.

XPS analysis revealed that the Ce^3+^/Ce^4+^ ratio was relatively consistent across all samples (0.6). This redox balance is critical because it has been suggested that the conversion of Ce^3+^ to Ce^4+^ contributes to oxidative stress, leading to bacterial cytotoxicity.^52^ Ce^4+^ is known to generate reactive oxygen species (ROS), which induce oxidative stress and damage bacterial membranes, proteins, and DNA. In contrast, Ce^3+^ exhibits an antibacterial effect, primarily through interactions with bacterial membranes rather than oxidative mechanisms.

The dual action of membrane disruption by Ce^3+^ and ROS-mediated oxidative stress by Ce^4+^ supports this antibacterial efficacy. In addition, clv-coated PGs exhibited enhanced antibacterial activity, which may be related to membrane disruption, facilitated cerium uptake, and oxidative stress, suggesting their potential to overcome bacterial resistance.

The cumulative release profile over 7 days shows that PGF-Ce0.2-clv releases more clv than the powder with the same composition, likely due to its fibrous morphology. Furthermore, incorporation of cerium ions into the phosphate glass network may increase the number of NBOs, creating additional interaction sites for clv and thereby enhancing its release.^30^ Cerium, in its +3 and +4 oxidation states, can form complexes with eugenol, the major component of clv, which possesses a hydroxyl (–OH) group and an ether (–O–) linkage, both of which can act as potential binding sites for cerium ions. In a previous work on Ga-loaded PGFs, Ga incorporation was also found to enhance clv release relatively to the Ga-free controls.^30^ However, advanced structural analysis is required to confirm this as FT-IR does not show big changes in the phosphate network with increasing Ce content.

Considering the application of PGPs and PGFs in wound healing, antibacterial and antioxidant testing were performed at the 24 h timepoint, where the critical need is early protection against oxidative stress and bacterial contamination during the initial healing phase.

PGPs and PGFs loaded with cerium and clv exhibited robust antioxidant capacity. Antioxidant activity was assessed through two approaches: combined chemical assays (DPPH and TPC assay) and evaluation of intracellular ROS levels. PG-clv glasses showed moderate radical scavenging (16–50% DPPH) and phenolic content (3–5 mg GAE g^−1^), with fibres outperforming powders. This activity is primarily driven by eugenol in clv, a phenolic compound that donates hydrogen to neutralise free radicals and terminate oxidative chain reactions.^53^

Introducing cerium dramatically increased this antioxidant activity (up to 87% DPPH, 25 mg GAE g^−1^). This enhancement arises from cerium oxide's SOD- and catalase-like behaviour; as described by Das *et al.*, cerium nanoparticles undergo reversible Ce^3+^/Ce^4+^ redox cycling to scavenge superoxide anions and decompose hydrogen peroxide, effectively “recycling” to provide sustained antioxidant protection.^54–56^ Notably, PGF-Ce-clv achieved higher DPPH and TPC values than the previously reported PGF-Ga-clv system, where Ga (0.5 mol% Ga_2_O_3_) provided only moderate antioxidant^30^

Consistent with the chemical assays, the intracellular ROS evaluation demonstrated an enhanced ability to scavenge intracellular ROS, while the PG-unl-clv and PG-Ce0.2-clv (both powder and fibre) induced no intrinsic oxidative stress. The significant reduction in fluorescence confirms that the complementary mechanisms of cerium (redox cycling) and clv (radical scavenging) act synergistically to shield cells from oxidative damage.

This sustained antioxidant release suggests these PGs can effectively reduce oxidative stress during wound healing. Because excessive ROS in the wound microenvironment is known to inhibit cell migration and sustain a pro-inflammatory state,^57^ the impact of this activity *via* an *in vitro* scratch assay was evaluated. Cells treated with the PGs dissolution products exhibited significantly faster migration rates compared to the control, achieving nearly complete wound closure by 48 h, which produced the highest closure (37%) compared with the corresponding unloaded PGs. This accelerated closure is attributed to the combined action of cerium and clv in scavenging excess ROS to support a pro-healing environment. Furthermore, accelerated tissue repair would be further supported by the inherent antibacterial effects of both cerium and clv, which work synergistically to protect the wound from infection-driven inflammation and delayed healing. These observations align perfectly with reports that cerium oxide nanoparticles enhance fibroblast migration by reducing ROS,^58^ and that eugenol reduces pro-inflammatory signalling to accelerate tissue remodelling.^59^

This work has demonstrated a facile and versatile route to multifunctional porous PGPs and PGFs that combine therapeutics effect and ion release, to achieve antibacterial effects, antioxidant activity, and cytocompatibility.

## 5. Conclusions

This study demonstrates the successful synthesis of porous PGPs and PGFs in the system P_2_O_5_–CaO–Na_2_O–Ce_2_O_3_ (Ce = 0, 0.1, 0.2 and 0.4 atomic%) *via* coacervation combined with supramolecular templating. Hierarchical porosity was achieved in PGPs and PGFs by adding the sacrificial surfactant P123. Structural characterization revealed that the amorphous nature is preserved upon calcination at 300 °C, temperature used to remove the porogen P123. XPS analysis demonstrated the presence of both Ce^3+^ and Ce^4+^ oxidation states, with Ce^3+^/Ce^4+^ ratio of 0.6 for PGP-CeX and PGF-Ce0.4.

Ion release studies showed that cerium addition increases the solubility of P and Ca from both PGPs and PGFs. Also, the incorporation of Ce was correlated with improved antibacterial properties against *E. coli.* Indirect MTT assays showed that PGFs were non-toxic to HaCaTs and exhibited higher cell viability than PGPs. The cytocompatibility of PGFs was further confirmed by live-cell staining of HaCaTs, which demonstrated extensive cell spreading across the fibre network. Coating PGP-Ce0.2 and PGF-Ce0.2 with clv improved the antioxidant properties, as demonstrated by DPPH and TPC assays.

The fibrous morphology of PGFs, together with Ce incorporation, promoted higher clv release in PGF-Ce0.2, resulting in enhanced antioxidant activity. This result was confirmed by DPPH and TPC assays, with PGF-Ce0.2-clv showing the strongest performance, reaching 87% radical scavenging activity and 25 mg GAE g^−1^. The antibacterial assay also confirmed that both PGP-Ce0.2-clv and PGF-Ce0.2-clv effectively inhibit *E. coli*.

The ROS assay confirmed that PG-unl-clv and PG-Ce0.2-clv (powder and fibre) do not induce oxidative stress and instead provide strong intracellular ROS scavenging. Consistent with these findings, the scratch assay revealed significantly enhanced cell migration and wound closure by 48 h, indicating that the cerium and clv combined antioxidant and antibacterial activities synergistically support a pro-healing environment and improved wound repair performance.

## Conflicts of interest

There are no conflicts to declare.

## Supplementary Material

TB-014-D5TB02254A-s001

## Data Availability

The data supporting this article have been included as part of the supplementary information (SI): optical image of PGPs and PGFs before calcination; XRD patterns of PGP-unl, PGP-Ce0.1, PGP-Ce0.2, PGP-Ce0.4, PGF-unl, PGF-Ce0.1, PGF-Ce0.2 and PGF-Ce0.4, SEM images of non-porous PGP-unl and PGF-unl; compositions of PGPs and PGFs expressed in oxide (mol%) measured *via* EDX; Ce 3d_3/2_ and Ce 3d_5/2_ XPS spectra of Ce^3+^ and Ce^4+^ for PGF-Ce0.4. See DOI: https://doi.org/10.1039/d5tb02254a.
