# Supplementary material for: Keratinocytes biocompatibility, antibacterial and antioxidant properties of porous coacervate phosphate glass fibres and powders loaded with cerium and clove oil: a comparative study
Source: J Mater Chem B. 2026 Mar 30;14(15):4771–89. doi: 10.1039/d5tb02254a (PMC13058909; doi:10.1039/d5tb02254a)
Supplement: TB-014-D5TB02254A-s001 [file TB-014-D5TB02254A-s001.pdf]

Supplementary Information (SI) for Journal of Materials Chemistry B.
This journal is © The Royal Society of Chemistry 2026

Supporting Information

Keratinocytes biocompatibility, antibacterial and antioxidant properties of porous coacervate phosphate glass fibres and powders loaded with cerium and clove oil: a comparative study

Zarrin Moghaddam [a], Rahul Sanwlani[b], Eveliny Tomas Nery [a,b], Irem Unalan [c], Oluwadunmininu Okude [b], Agron Hoxha[a], Charlotte A. Berry, [a] Kavin Hettiarachchilage, [a] Steven J. Hinder [e], Mark A. Baker[e], Monica Felipe-Sotelo[a], Alessandra Pinna [f, d], Jorge Merino-Gutierrez [b], Aldo R. Boccaccini [c*], Patrizia Camelliti [b*], Daniela Carta [a*]

[a] *School of Chemistry and Chemical Engineering, University of Surrey, Guildford GU2 7XH, United Kingdom*

[b] *School of Biosciences, University of Surrey, Guildford GU2 7XH, United Kingdom*

[c] *Institute of Biomaterials, Department of Materials Science and Engineering, Friedrich-Alexander-University Erlangen-Nuremberg, Caustraße 6, 91058 Erlangen, Germany*

[d] *School of Veterinary Medicine, Faculty of Health and Medical Sciences, University of Surrey, Guildford GU2 7XH, United Kingdom*

[e] *The Surface Analysis Laboratory, Engineering and Physical Sciences, University of Surrey, GU2 7XH, Guildford, United Kingdom*

[f] *Department of Materials, Imperial College London, SW7 2AZ, London, United Kingdom*

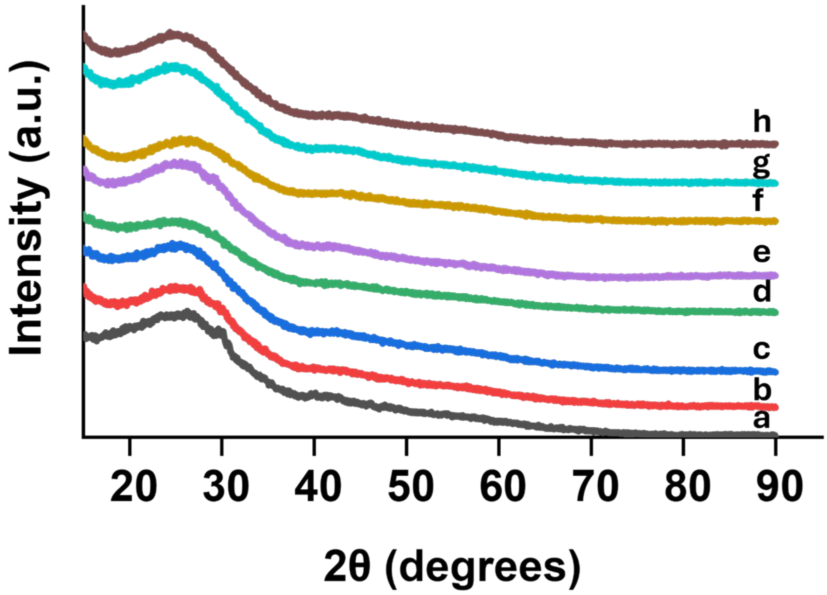

Figure SI_1. *XRPD patterns of a) PGP-unl, b) PGP-Ce0.1, c) PGP-Ce0.2, d) PGP-Ce0.4, e) PGF-unl, f) PGF-Ce0.1, g) PGF-Ce0.2, h) PGF-Ce0.4*

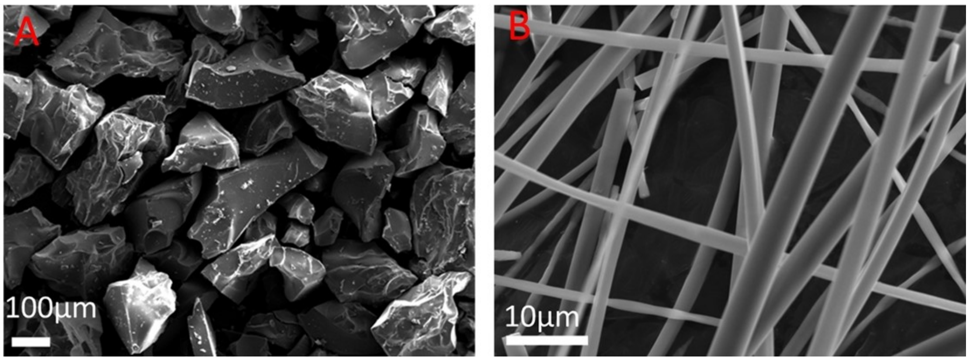

Figure SI_2. *SEM images of non-porous A) PGP-unl, B) PGF-unl*

Table SI_1. *Compositions of PGPs and PGFs in terms of oxide (mol %) measured via EDX.*

**Samples**	**Oxide (mol %)**				
	P_2O_5	CaO	Na_2O	Ce_2O_3	CeO_2
**PGP-unl**	47.8±1.0	43.6±0.5	8.6±0.4	-	-
**PGP-Ce0.1**	48.2±0.7	41.5±1.0	10.0±0.7	0.07±0.01	0.23±0.01
				-	
**PGP-Ce0.2**	46.8±1.0	42.6±0.5	10.0±0.4	0.15±0.01	0.48±0.1
**PGP-Ce0.4**	48.3±0.6	39.9±1.0	10.5±0.7	0.35±0.03	1.0±0.2
**PGF-unl**	50.8±2.0	40.8±1.0	8.4±0.4	-	-
**PGF-Ce0.4**	47.5±0.7	43.1±1.0	8.5±0.3	0.21±0.02	0.7±0.1

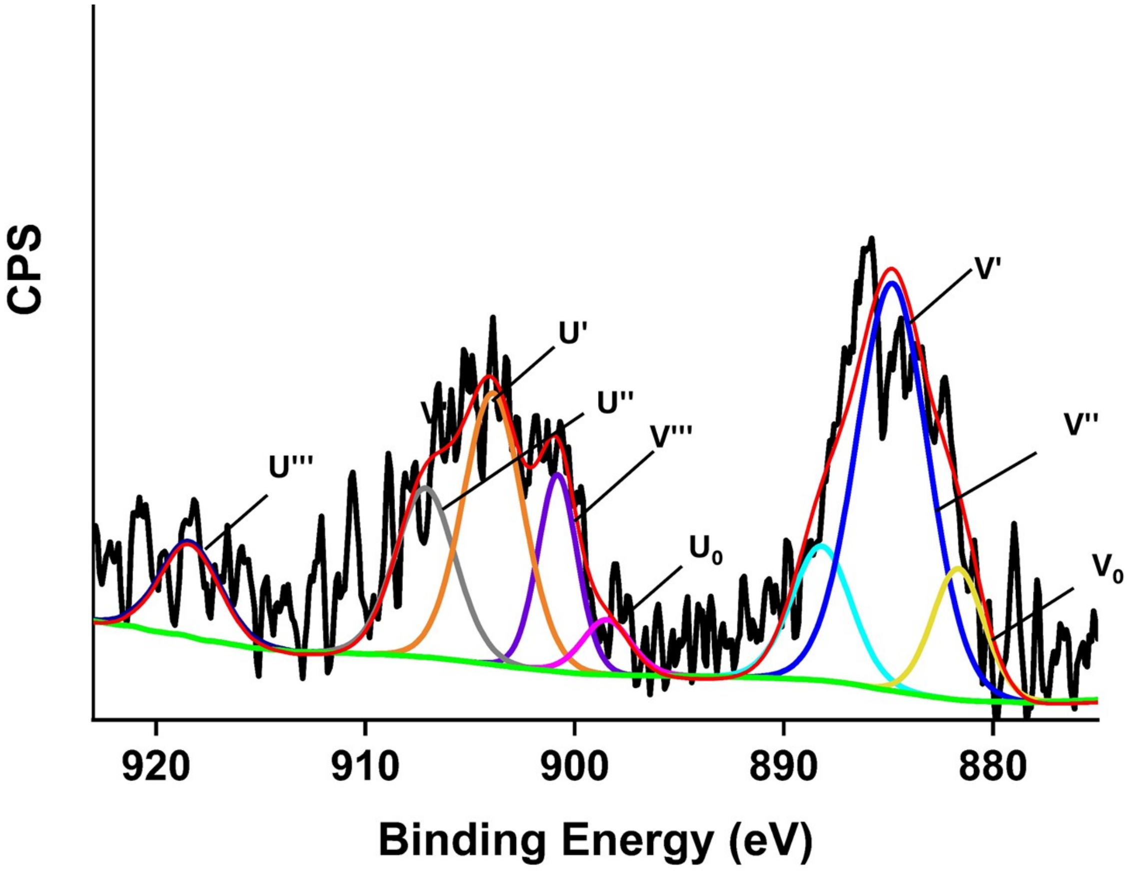

Figure SI_3. *Fitted Ce 3d$_{3/2}$ and Ce 3d$_{5/2}$ XPS spectra of Ce^{3+} and Ce^{4+} for PGF-Ce0.4*

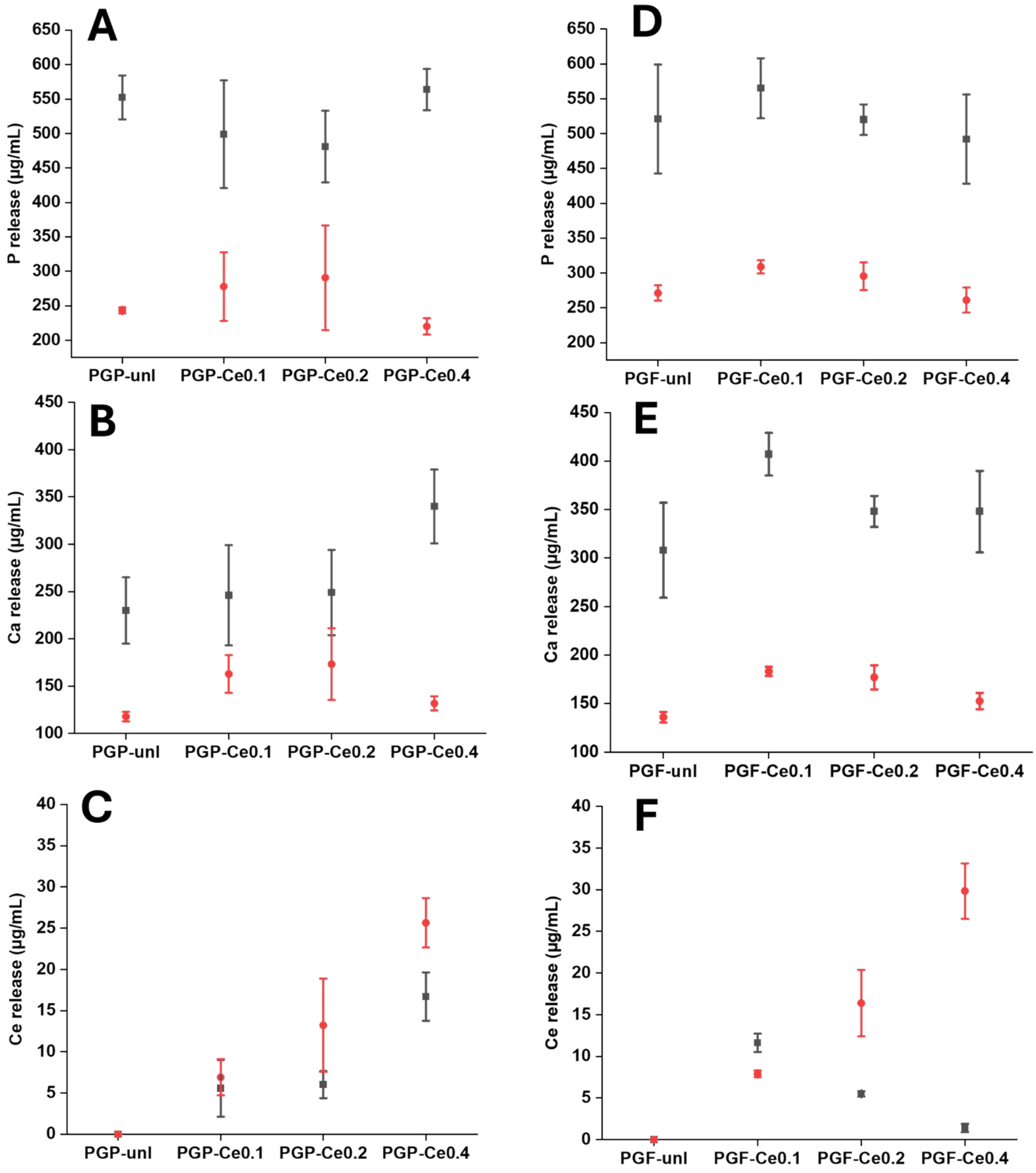

Figure SI_4. *Release of P, Ca and Ce from PGPs (A-C) and PGFs (D-F) after immersion in PBS (black) and DI water (red) after3 h. Error bars indicate the mean ± standard deviation (n = 3)*

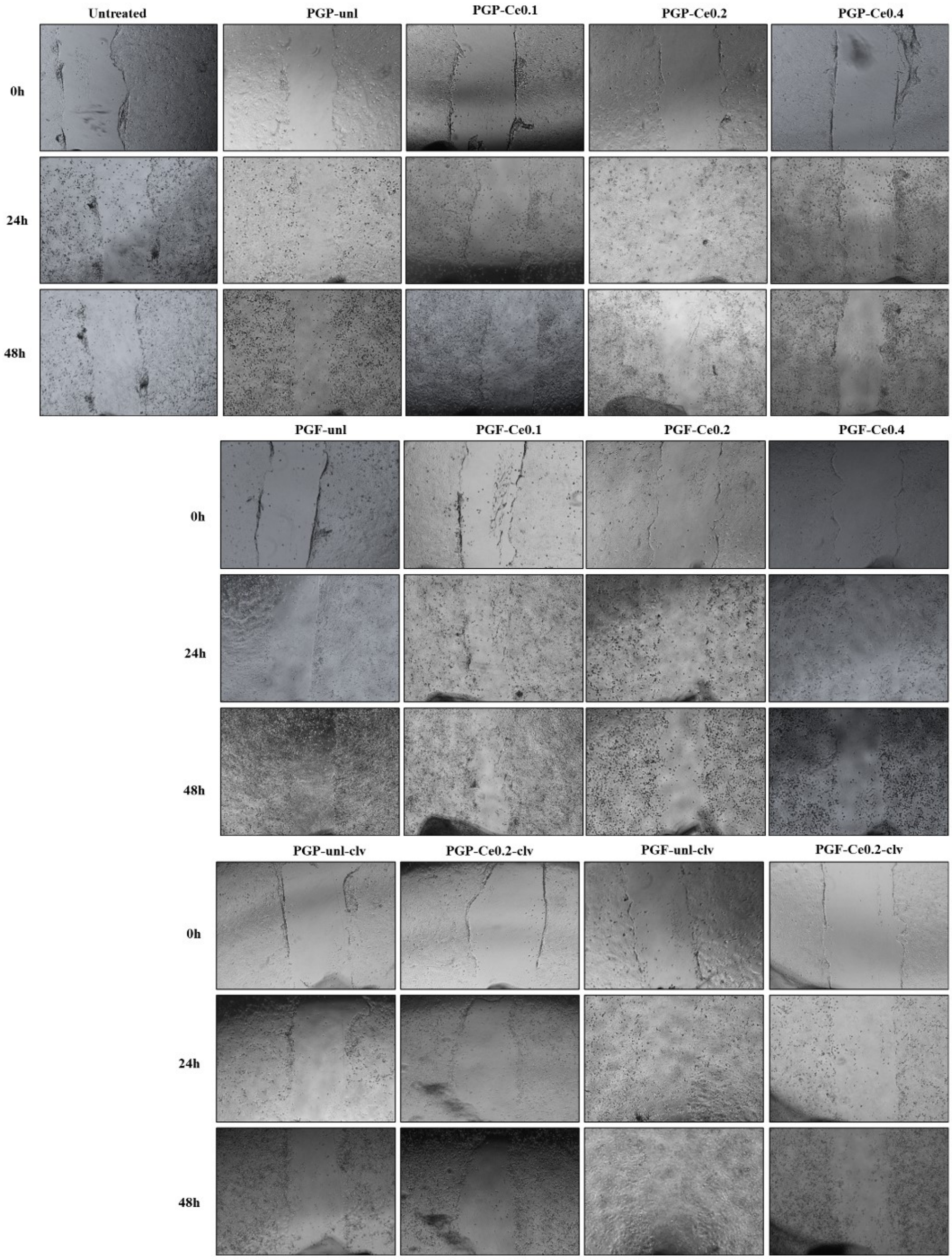

Figure SI_5. *Representative micrographs showing scratch area closure over time for different treatments*
